# Differential Expression of Immune Response Genes in Asymptomatic Chronic Chagas Disease Patients *Versus* Healthy Subjects

**DOI:** 10.3389/fcimb.2021.722984

**Published:** 2021-09-06

**Authors:** Inmaculada Gómez, M. Carmen Thomas, Génesis Palacios, Adriana Egui, Bartolomé Carrilero, Marina Simón, Basilio Valladares, Manuel Segovia, Emma Carmelo, Manuel Carlos López

**Affiliations:** ^1^Instituto de Parasitología y Biomedicina López-Neyra, Consejo Superior de Investigaciones Científicas, Granada, Spain; ^2^Instituto Universitario de Enfermedades Tropicales y Salud Pública de Canarias, Universidad de La Laguna, La Laguna, Spain; ^3^Unidad Regional de Medicina Tropical, Hospital Universitario Virgen de la Arrixaca, Murcia, Spain; ^4^Departamento de Obstetricia y Ginecología, Pediatría, Medicina Preventiva y Salud Pública, Toxicología, Medicina Legal y Forense y Parasitología, Universidad de La Laguna, La Laguna, Spain

**Keywords:** chronic Chagas disease, *Trypanosoma cruzi*, transcriptional profiling, high-throughput RT-qPCR, immunological pathway, biomarkers, indeterminate form

## Abstract

Infection by the *Trypanosoma cruzi* parasite causes Chagas disease and triggers multiple immune mechanisms in the host to combat the pathogen. Chagas disease has a variable clinical presentation and progression, producing in the chronic phase a fragile balance between the host immune response and parasite replication that keeps patients in a clinically silent asymptomatic stage for years. Since the parasite is intracellular and replicates within cells, the cell-mediated response of the host adaptive immunity plays a critical role. This function is mainly orchestrated by T lymphocytes, which recognize parasite antigens and promote specific functions to control the infection. However, little is known about the immunological markers associated with this asymptomatic stage of the disease. In this large-scale analysis, the differential expression of 106 immune system-related genes has been analyzed using high-throughput qPCR in *T. cruzi* antigen-stimulated PBMC from chronic Chagas disease patients with indeterminate form (IND) and healthy donors (HD) from endemic and non-endemic areas of Chagas disease. This analysis revealed that there were no differences in the expression level of most genes under study between healthy donors from endemic and non-endemic areas determined by PCA and differential gene expression analysis. Instead, PCA revealed the existence of different expression profiles between IND patients and HD (*p* < 0.0001), dependent on the 32 genes included in PC1. Differential gene expression analysis also revealed 23 upregulated genes (expression fold change > 2) and 11 downregulated genes (expression fold change < 0.5) in IND patients *versus* HD. Enrichment analysis showed that several upregulated genes in IND patients participate in relevant immunological pathways such as antigen-dependent B cell activation, stress induction of HSP regulation, NO2-dependent IL12 pathway in NK cells, and cytokine-inflammatory response. The antigen-specific differential gene expression profile detected in these patients and the relevant immunological pathways that seem to be activated could represent potential biomarkers of the asymptomatic form of Chagas disease, helpful to diagnosis and infection control.

## Introduction

Chagas disease, also known as American trypanosomiasis, is caused by the protozoan parasite *Trypanosoma cruzi* and is considered one of the most prevalent neglected tropical diseases. It affects around 6–7 million people worldwide, causing approximately 20,000 deaths annually (World Health Organization, 2020). Although it is endemic in Latin America, the migratory movements have changed the epidemiological profile of this infection, representing a serious global health problem today (Schmunis and Yadon, 2010; World Health Organization, 2020).

The clinical course of Chagas disease is characterized by an acute and a chronic phase of infection. The acute phase usually subsides spontaneously after which, if left untreated, the patient will still be chronically infected (Pérez-Molina and Molina, 2018). The reason for this fact is that the parasite spreads from the blood to the tissue that remains hidden, which makes them less accessible for the immune response (Tarleton, 2001). In the chronic phase, most patients are asymptomatic, without developing any clinical symptoms or signs. This is possible due to the existence of a fragile balance between parasite replication and host immune response, which may cause patients to remain clinically silent for a period of 10 to 25 years (Dos Santos Virgilio et al., 2014). This is known as the indeterminate phase of Chagas disease, characterized by seropositivity for *T. cruzi* and absence of cardiac or digestive symptoms with normal electrocardiography and radiography of the chest, esophagus, and colon, all responsible for a good prognosis of the disease in these patients (Pinto Dias, 1989; World Health Organization, 2020). The imbalance between the immune system response and parasite replication is crucial for the disease progression. Thus, 30%–40% of chronic patients eventually develop a symptomatic phase (Rassi et al., 2010). About 30% of them show cardiac alterations, and up to 10% show digestive megasyndromes and/or neurological disorders (Rassi et al., 2010). The most severe cases of cardiac alterations lead to chronic chagasic cardiomyopathy, associated with high mortality rates in Chagas disease patients (Morris et al., 1990; Rocha et al., 2003).

The diagnosis of Chagas disease is well defined. Specifically, in the chronic phase, serological techniques such as indirect immunofluorescence (IFA), indirect hemagglutination (HAI), and enzyme-linked immunosorbent assay (ELISA) are applied for the detection of antibodies against the parasite (Fife and Muschel, 1959; Camargo, 1966; Camargo et al., 1971; Voller et al., 1975). However, at present, the development of biomarkers of pathology or disease progression remains a necessity, which would represent a major achievement toward improving the clinical management of patients with Chagas disease (Balouz et al., 2017).

The pathogenesis of chronic Chagas disease is currently considered to be multifactorial. In the course of this phase, in addition to other factors, such as the virulence of the *T. cruzi* strain and tissue tropism, inflammation is the main determinant of the disease progression (Machado et al., 2012; Dutra et al., 2014; Poveda et al., 2014).

The host’s defense reaction against the parasite involves mechanical effectors of the innate and adaptive immune response (Tarleton, 2007), which is mainly characterized by processes of cell proliferation, production of cytokines, and induction of cell death mechanisms (De Meis et al., 2009). During the evolution of Chagas disease, cellular responses are crucial. Thus, it has been demonstrated that chronic patients (both indeterminate and cardiac individuals) present in their bloodstream high frequencies of activated T cells (Dutra et al., 1994). While in the chronic cardiac form an inflammatory environment predominates with the production of cytokines such as TNFα, IFNγ, and other cytotoxic molecules involving CD8^+^ T cells, in the chronic indeterminate form, a regulatory immune response, characterized by interleukin 10 and interleukin 17 production, predominates (Pérez-Molina and Molina, 2018). Nevertheless, elevated levels of TNFα and IFNγ have also been detected in IND patients compared to healthy subjects (Ferreira et al., 2003; Requena-Méndez et al., 2013). However, there is controversy and other authors have described an opposite correlation between the expression of *IFNγ* and cardiac disease (Laucella et al., 2004). Besides, in asymptomatic patients, *T. cruzi* antigen-specific co-production of TNFα, IFNγ, and IL2 cytokines by CD8^+^ T cells has been found in high proportion which decreases as the disease progresses toward cardiac forms (Mateus et al., 2015). On the other hand, it has been shown that circulating activated T cells in asymptomatic and symptomatic subjects express both inflammatory and anti-inflammatory cytokines, which is consistent with active immunoregulation in the chronic phase (Dutra et al., 1997; Cunha-Neto et al., 2005).

Given that the loss of balance between the immune system response and parasite replication existing in asymptomatic patients is crucial for the disease progression, to know the immune mechanisms that lead to the control of the establishment of the infection and its progression is a priority.

The aim of this work was to elucidate the gene expression patterns that are involved in chronic Chagas disease patients with indeterminate form (IND) of the infection. For this purpose, a high-throughput qPCR was used to analyze, at the same time, the expression level of 106 immune system-related genes in human peripheral blood mononuclear cell (PBMC) samples from IND patients and compared to that from healthy donors coming from non-endemic (HDc) and endemic areas (HDe) of the disease. The results have provided a large collection of differential gene expression data in IND patients *versus* healthy donors. Comparative analyses of the differentially expressed genes among IND, HDc, and HDe subjects have allowed us to the identification in chronic indeterminate patients of antigen-specific differential gene expression patterns that involve a large set of immune-related genes which participate in several relevant immunological pathways. Study of the differential expression of these genes and the immune routes in which they are involved will improve our knowledge in the establishment of the *T. cruzi* infection and could also represent new potential biomarkers of the asymptomatic stage in Chagas disease patients.

## Material and methods

### Ethical Considerations

The protocols used in this study were approved by the Ethics Committees of the Consejo Superior de Investigaciones Científicas (Spain—Reference: 094/2016) and of the Hospital Virgen de la Arrixaca (Murcia, Spain—Reference: MTR-05/2016). The participation of all patients and healthy donors included in this study was completely voluntary, and furthermore, a signed informed consent form was obtained from each of them before their inclusion.

### Study Cohort

The adult chronic Chagas disease patients originally from endemic areas and residents of Spain included in this study were recruited, diagnosed, and clinically evaluated in the Hospital Virgen de la Arrixaca from Murcia (Spain). These patients, who had not received antiparasitic treatment, were diagnosed out according to the WHO criteria based on two conventional serological tests (Chagas ELISA, Ortho Clinical Diagnostics, and Inmunofluor Chagas, Biocientífica, Argentina) and characterized as indeterminate (IND) patients due to the absence of cardiac (G0 following Kuschnir classification) or digestive manifestations (**Supplementary Table 1**). In addition, healthy donors from endemic (n = 14) and non-endemic areas (n = 20) were included in this study. The data referring to the age, sex distribution, and country of origin of each of the subjects included in this study are detailed in **Table 1**.

**Table 1:** Epidemiological and demographic data of the study cohort.

Patient group		Origin (%)	Age (years)		Sex [% female (F)/male (M)]
			Mean (± SD)	Range	
**Healthy donors**	**From non-endemic area** (n = 20)	100% Spain	37.6 (12.8)	22–56	60% F
					40% M
	**From endemic area** (n=14)	28.6% Colombia	37.5 (8.4)	21–54	64.3% F
		14.3% Venezuela			
		7.1% Chile			
		7.1% Panama			35.7% M
		7.1% Ecuador			
		35.7% ND			
**Indeterminate patients** (n=71)		93% Bolivia	36.7 (9.6)	18–59	70.9% F
		1.2% Salvador			
		1.2% Paraguay			29.1% M
		4.7% ND			

ND, Not determined.

In this study, a total of 39 samples from 71 IND patients and 30 samples from 34 healthy donors were included. Due to the quantity of RNA required to carry out the cDNA synthesis for high-throughput RT-qPCR and the limited number of cells isolated from the blood sample of particular patients, in some cases it was necessary to mix cells from some patients. The collection of new blood samples was not possible in any case since the patients with Chagas disease were treated immediately after being diagnosed. Thus, into the cohort of IND patients, 15 samples corresponded to individual samples (38.5%), 16 to a mixture of 2 patients (41%), and 8 to a mixture of 3 patients (20.5%). In the case of healthy donors, 20 samples from subjects from non-endemic areas were tested (all of them individual samples) together to 10 samples of donors from endemic areas, 6 of which corresponded to individual samples (60%) and 4 to a mixture of 2 subjects (40%).

### Isolation of Peripheral Blood Mononuclear Cells

Thirty milliliters of peripheral blood from subjects was aseptically collected through venipuncture into EDTA-coated tubes. Peripheral blood mononuclear cells (PBMCs) were isolated 16–18 h after blood collection by density gradient centrifugation using Lymphoprep™ (Axis Shield) following the previously described protocol (Marañón et al., 2011). The purified PBMCs were suspended in heat-inactivated fetal bovine serum (iFBS) with 10% DMSO and cryopreserved in liquid nitrogen until use.

### Isolation of *T. cruzi* Soluble Antigens (*Tc*SA)

*T. cruzi* (SOL strain) soluble antigens (*Tc*SA), employed to perform *in vitro* stimulation of PBMCs from patients and healthy donors, were extracted as previously described (Egui et al., 2017). Briefly, mycoplasma-free rhesus monkey kidney epithelial cells (LLC-MK2 line; CCL-7, Manassas, VA, USA) were cultured at a concentration of 4×10^4^ cells/cm^2^ in T-75-cm^2^ tissue culture flasks (Falcon) with RPMI-1640 medium (Gibco, Life Technologies), supplemented with 2 mM L-glutamine (Gibco), 10% iFBS, and 50 µg/ml gentamicin (Thermo Fisher Scientific) at 37°C in a humidified atmosphere containing 5% CO_2_. The semi-confluent monolayer of cultured cells was infected with highly infective trypomastigote forms of the *T. cruzi* SOL strain (MHOM/ES/2008/SOL; DTU V) isolated from *T. cruzi*-infected mice, at a parasite:cell ratio of 4:1 for 12 h. After 96–120 h of infection, collection of the trypomastigote and amastigote forms present in the infected LLCMK-2 cell culture supernatants began. The recovered trypomastigote and amastigote forms were collected at 1,258 rcf and washed twice with phosphate-buffered saline (PBS 1×), Subsequently, these parasites were resuspended, at a ratio of 1:1 (trypomastigote: amastigote) and a density of 1×10^6^ parasites/μl in lysis buffer (50 mM Tris–HCl at pH 7.4, 0.05% Nonidet P-40, 50 mM NaCl, 1 mM phenylmethylsulfonyl fluoride (PMSF), 1 μg/ml leupeptin), and sonicated three times with pulses of 50–62 kHz for 40 s with time intervals of 20 s. Finally, the soluble total protein extracts were obtained after centrifugation at 6,700 rcf for 20 min at 4°C.

The protein concentration of the extract was determined using a micro bicinchoninic acid (BCA) protein assay kit (Thermo Scientific, Waltham, MA, USA), and the protein profile was analyzed by SDS-PAGE after Coomassie blue staining. The antigenic and immunogenic capacities of *Tc*SA were tested by ELISA and in lymphoproliferation assays using frozen splenocytes from *T. cruzi* chronically infected mice.

### Thawing and Stimulation of Peripheral Blood Mononuclear Cells

Cryopreserved PBMCs were thawed in a water bath at 37°C; transferred to a 15-ml Falcon tube containing 10 ml of RPMI-1640 with 2 mM L-glutamine, 10% iFBS, and 50 µg/ml of gentamicin; and centrifuged at 453 rcf for 10 min. After centrifugation, the PBMCs were again suspended in 2 ml of supplemented RPMI-1640 medium and the cell number was determined by manual counting using Trypan blue exclusion assay. Subsequently, all PBMC samples were plated in 12-well plates at concentrations of 7.5–8.5×10^6^ cells/ml in a maximum volume of 3.5 ml/well and cultured for 4 h in an incubator at 37°C in 5% CO_2_ to allow equilibration of basal gene expression under *in vitro* growth conditions. Finally, the PBMCs from IND and HD were stimulated with *Tc*SA (10 μg/ml) and cultured for 14–14.5 h at 37°C in 5% CO_2_.

### RNA Isolation, Quantification, and Quality Analysis

Total RNA isolation from stimulated PBMCs was performed using the RNeasy Plus Mini Kit (Qiagen), eliminating the genomic DNA and obtaining mRNA enrichment samples following the manufacturer’s instructions. The extracted RNA was quantified by spectrophotometry (NanoDrop 1000, Thermo Fisher Scientific) and confirmed by fluorometry (Qubit, Invitrogen). The quality of the RNA was determined by analyzing its integrity by microfluidic chip electrophoresis (2100 Bioanalyzer, Agilent Technologies). All samples showed to have an RIN (RNA integrity number) between 8 and 10. The samples were stored at -80°C until use.

### Reverse Transcription and High-Throughput Real-Time Quantitative PCR

Two micrograms of total RNA of each patient sample was used for cDNA synthesis by reverse transcription using the High-Capacity cDNA Reverse Transcription Kit (Applied Biosystems) following the manufacturer’s instructions. The resulting cDNA samples were stored at -20°C until use. High-throughput RT-qPCR was performed using QuantStudio™ 12K Flex Real-Time PCR System (Thermo Fisher Scientific) according to the manufacturer’s protocol, as indicated in Hernandez-Santana et al. (2016). Custom TaqMan OpenArray Real-Time PCR Plates included 112 Gene Expression Assays organized in 48 sub-arrays. All primers and probes were commercially available by Thermo Fisher Scientific. The complete list of genes and the corresponding probes mapping in each gene are shown in **Supplementary Table 2**. All reactions were performed in triplicate. Cq values produced by this platform are already corrected for the efficiency of the amplification (Hernandez-Santana et al., 2016).

### Data Analysis and Statistics

The arithmetic average quantitative cycle (Cq) was used for data analysis. The Cq values for each qPCR run were exported from QuantStudio™ 12K Flex Real-Time PCR System, as Excel files, and imported into GenEx software (v.6, MultiD). The expression stability of the candidate reference genes (RGs) was evaluated using RefFinder (Xie et al., 2012) (heartcure.com.au), which integrates the algorithms GeNorm (Vandesompele et al., 2002), NormFinder (Andersen et al., 2004), and BestKeeper (Pfaffl et al., 2004), as well as the comparative ΔCt method (Silver et al., 2006). Three genes showed the most stable expression (*STAT3*, *IL10RA*, and *IFNAR*) (all with GeNorm M-value < 0.5) and were used for normalization to obtain normalized relative quantities (NRQ).

The GraphPad Prism statistical package version 8 (GraphPad Software Inc., San Diego, CA, USA) and SPSS software (SPSS Inc., Chicago, IL, USA) were used to perform statistical analyses. Kolmogorov–Smirnov and Shapiro–Wilk tests (α=0.05) were used to check data normality, and statistical significance was determined by a two-tailed Mann–Whitney test or a two-tailed unpaired t-test, as appropriate, considering *p* < 0.05 as statistically significant.

Differentially expressed genes between healthy donors from endemic (HDe) and non-endemic areas (HDc) of Chagas disease, as well as between chronic Chagas disease patients with indeterminate form (IND) and healthy donors (HD), were identified using two parameters: the fold change of gene expression (FC) and the statistical significance (*p*-value). FC was calculated as the ratio between biological groups (HDc and HDe, IND and HD, or individual samples from IND patients and HD) and expressed as log_2_. To display changes, volcano plots were made by plotting the –log_10_ *p-*value (determined by a two-tailed unpaired t-test) on the y-axis, and log_2_ of FC on the x-axis. Genes passing both biological significance threshold (log_2_ of FC > 1 or < −1, corresponding to FC > 2 or < 0.5) and statistical significance threshold (–log_10_ *p* > 1.3, corresponding to *p* = 0.05 and (–log_10_ *p* > 3, corresponding to *p* = 0.001) were marked in red and blue, attending to their upregulation and downregulation, respectively. Those genes were considered biologically relevant and used for further biological interpretation. An interaction network between differentially expressed genes in IND *versus* HD was generated using the Retrieval of Interacting Genes Database (STRING) v.11 (Szklarczyk et al., 2019) available at https://string-db.org/. Active interactions sources, including experiments, databases, co-occurrence, gene fusion, neighborhood, and co-expression as well as species limited to “Homo sapiens,” and an interaction score > 0.9 were applied to construct PPI networks.

Principal component analysis (PCA) was applied for multivariate analysis on NRQ values to determine the structure of the dataset. Differences in scores of plotted principal components between the groups were confirmed by a two-tailed Mann–Whitney test or a two-tailed unpaired t-test, depending on data that had not or had a normal distribution, respectively, using SPSS 25 (SPSS Inc., Chicago, IL, USA).

### Enrichment Analysis

To further investigate on the potential biological processes and pathways involved in chronic Chagas disease indeterminate form (IND), gene set enrichment analysis was performed using the GSEA 4.1.0 computational method (Mootha et al., 2003; Subramanian et al., 2005). Canonical pathway gene sets derived from the BioCarta pathway database included in *C2: curated gene sets collection in Molecular Signatures Database (MSigDB)* were used for the analysis (Subramanian et al., 2005; Liberzon et al., 2011) [parameters set for GSEA were as follows: permutations = 100,000, permutation type: phenotype (sample n > 7), enrichment statistic: weighted, metric for ranking genes: t-test, max size: 500, min size: 3].

## Results

To improve the knowledge of the specific immune response generated during infection in Chagas disease patients, the gene expression pattern of particular genes involved in the immune response elicited after *T. cruzi* infection has been analyzed in these patients. Thus, expressions of 106 immune system-related genes in response to *T. cruzi* proteins have been determined in human PBMCs from Chagas disease patients at the indeterminate phase of the disease (n = 39) together to those from healthy donors from endemic (n = 10) and non-endemic (n = 20) areas of Chagas disease. Total RNA isolated from *T. cruzi* soluble antigen-stimulated PBMCs from IND and HD subjects was used for cDNA synthesis followed by high-throughput RT-qPCR of triplicates, to obtain an arithmetic average quantitative cycle (Cq) useful for comparative analyses between patients and healthy donors. The expression stability of all the included genes was also evaluated. Since the *STAT3*, *IL10RA*, and *IFNAR* genes showed the most stable expression values (with GeNorm M-values < 0.5), they were used as reference genes (RGs) for normalization of the data set to obtain normalized relative quantities (NRQ).

### Comparative Analysis of the Gene Expression Profile Between Healthy Subjects

To determine whether there were differences in gene expression level among the healthy donors (HD) related to their origin, the NRQ values obtained for the 106 analyzed genes in subjects from endemic (HDe) and non-endemic areas (HDc) of Chagas disease were analyzed and compared employing GenEx software. As observed in the heatmap plot shown in **Supplementary Figure 1**, no differences were observed in the gene expression values between subjects from endemic and non-endemic countries since the different clusters generated by the software included HDe and HDc subjects indistinctly.

**Figure 1:**
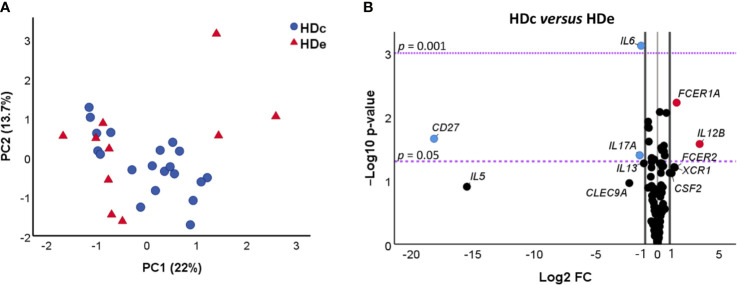
Comparative analysis of gene expression of 106 genes in healthy donors from non-endemic (HDc, n = 20) and endemic (HDe, n = 10) areas of Chagas disease. **(A)** Principal component analysis (PCA) of NRQ (normalized relative quantities) values of gene expression of 106 analyzed genes in HDc (blue circles) and HDe (red triangles). Principal components 1 (PC1) and 2 (PC2) are plotted on the x and y axes, respectively, and the proportion of variance captured for both components is given as a percentage. **(B)** Volcano plot of the differential expression level of the 106 analyzed genes between samples of HDc and HDe subjects. The x-axis represents log_2_ of the expression fold change between HDc and HDe (Log_2_ FC), where FC is calculated as the ratio between two groups (HDc/HDe). The y-axis corresponds to the statistical significance, expressed as the negative logarithm of the *p*-value (-Log_10_ *p*-value). The purple horizontal lines indicate the cutoffs for the statistical significance values *p* = 0.05 and *p* = 0.001. The black vertical lines represent the log_2_ of FC of −1 and 1 (corresponding to FC of 0.5 and 2, respectively) which were used as biological thresholds to identify differentially expressed genes. The negative values correspond to downregulated genes (blue dots) and the positive values to the upregulated genes (red dots). Black dots comprising between the established thresholds represent non-differentially expressed genes between HDc and HDe.

The NRQ values from 106 genes of HDe and HDc subjects were also employed to determine the structure of the dataset by principal component analysis (PCA). The obtained results plotted in **Figure 1A** indicated that principal component 1 (PC1) and principal component 2 (PC2) accounted for 22% and 13.7% of the variance among the individuals, respectively. As observed, HDe and HDc did not exhibit differences on gene expression values of the genes under study (**Figure 1A**) as they presented a very similar distribution and were not separated by the principal components. These results were confirmed by a two-tailed Mann–Whitney test or a two-tailed unpaired t-test, as appropriate, showing that there were no statistically significant differences between the scores obtained in the two groups for each component (PC1 *p* = 0.65, PC2 *p* = 0.23).

A third differential gene expression analysis based on the fold change of gene expression (FC) and its statistical significance was further carried out to elucidate whether there were any differences in the gene expression level of particular genes between the healthy donors coming from endemic areas and those from non-endemic regions. The obtained results, represented in a volcano plot (**Figure 1B**) to illustrate both significance and magnitude of the changes, showed that 5 out of the 106 studied genes were differentially expressed in the HDe *versus* HDc group (log_2_ fold change (FC) > 1 or < -1) with statistical significance (*p* < 0.05). Two genes (*FCER1A* and *IL12B*) were found to be upregulated in HDc when compared with the HDe subjects (log_2_ FC > 1, corresponding with a greater than two-fold change). On the contrary, three genes (*CD27*, *IL6*, and *IL17A*) were downregulated in HDc *versus* HDe subjects (log_2_ FC < -1, corresponding with a less than half fold change).

Altogether, these results indicate that there were no significant differences in the expression level of 95.3% of the genes under study between healthy donors coming from endemic areas and those living in non-endemic regions, suggesting that they could be considered as a single group of healthy donors (HD). Despite that, the five genes differentially expressed in HDc and HDe were also analyzed considering HDe and HDc as independent groups of subjects.

### Identification of Genes Differentially Expressed in IND and Healthy Subjects

**Figure 2:**
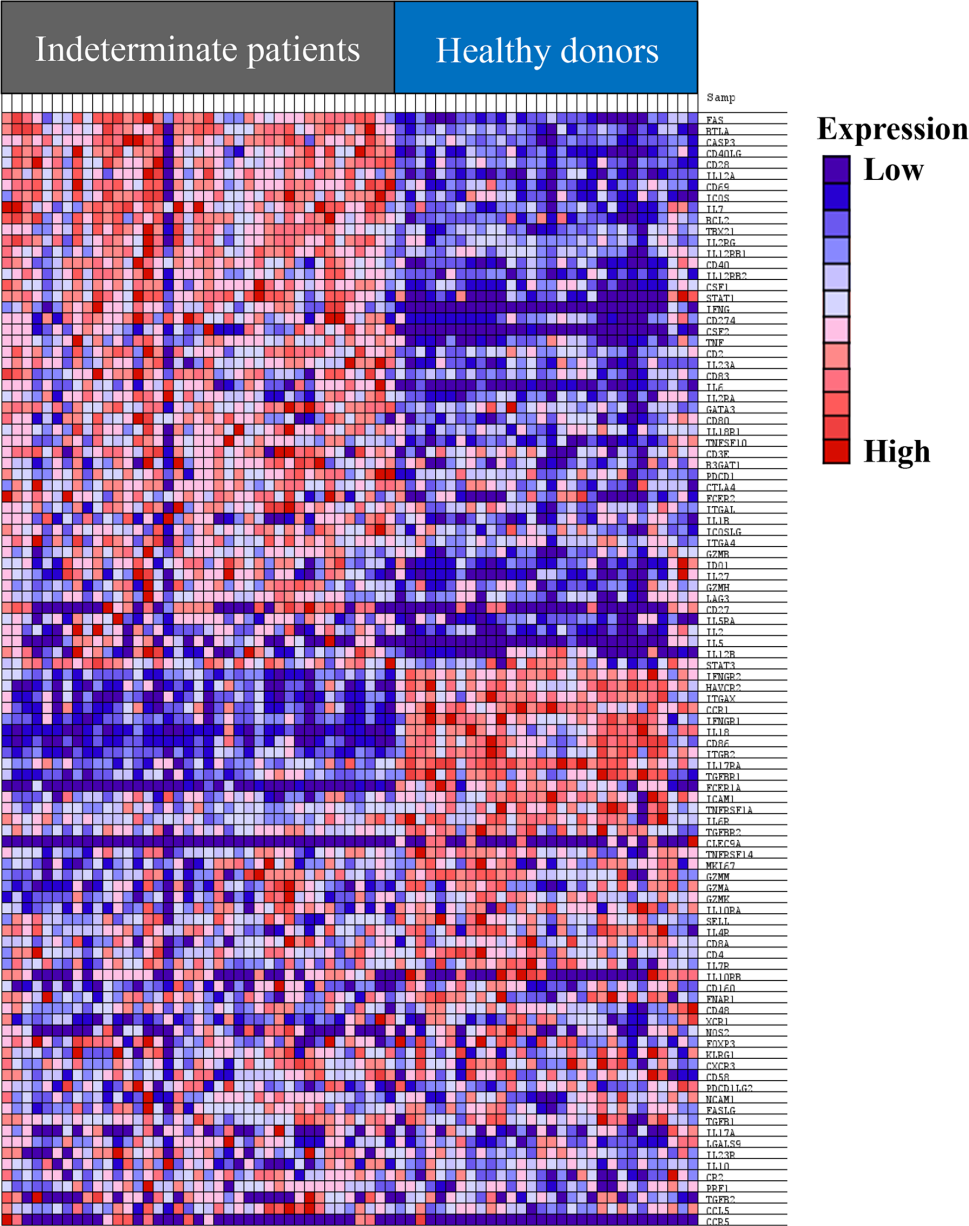
Heat map of the top 100 genes determined by GSEA analysis in IND and HD subjects. The values of the gene expression level of each gene are represented as colors, ranging from dark red to dark blue, based on the highest and lowest normalized relative quantities (NRQ) values of each gene, respectively. The genes represented in vertical order from the top to the bottom are *FAS*, *BTLA*, *CASP3*, *CD40LG*, *CD28*, *IL12A*, *CD69*, *ICOS*, *IL7*, *BCL2*, *TBX21*, *IL2RG*, *IL12RB1*, *CD40*, *IL12RB2*, *CSF1*, *STAT1*, *IFNG*, *CD274*, *CSF2*, *TNF*, *CD2*, *IL23A*, *CD83*, *IL6*, *IL2RA*, *GATA3*, *CD80*, *IL18R1*, *TNFSF10*, *CD3E*, *B3GAT1*, *PDCD1*, *CTLA4*, *FCER2*, *ITGAL*, *IL1B*, *ICOSLG*, *ITGA4*, *GZMB*, *IDO1*, *IL27*, *GZMH*, *LAG3*, *CD27*, *IL5RA*, *IL2*, *IL5*, *IL12B*, *STAT3*, *IFNGR2*, *HAVCR2*, *ITGAX*, *CCR1*, *IFNGR1*, *IL18*, *CD86*, *ITGB2*, *IL17RA*, *TGFBR1*, *FCER1A*, *ICAM1*, *TNFRSF1A*, *IL6R*, *TGFBR2*, *CLEC9A*, *TNFRSF14*, *MKI67*, *GZMM*, *GZMA*, *GZMK*, *IL10RA*, *SELL*, *IL4R*, *CD8A*, *CD4*, *IL7R*, *IL10RB*, *CD160*, *FNAR1*, *CD48*, *XCR1*, *NOS2*, *FOXP3*, *KLRG1*, *CXCR3*, *CD58*, *PDCD1LG2*, *NCAM1*, *FASLG*, *TGFB1*, *IL17A*, *LGALS9*, *IL23R*, *IL10*, *CR2*, *PRF1*, *TGFB2*, *CCL5*, and *CCR5*.

Next, we compared the expression levels of the 106 genes in IND and HD patients. As a first approach, gene set enrichment analysis (GSEA) was performed using the GSEA 4.1.0 computational method (Mootha et al., 2003; Subramanian et al., 2005) using the NRQ of genes from IND and HD. **Figure 2** shows a heat map from the top 100 genes in IND and HD groups and it reveals a clear difference in the pattern of gene expression between the groups. Thus, more than half of the genes (at least 49 of the genes) were overexpressed in most IND patients (**Figure 2**). Furthermore, the expression level of 11 genes was significantly reduced in the majority of IND patients and other 11 genes showed to be downregulated in many IND patients (**Figure 2**). Specifically, the expression levels of the *IL18*, *CD86*, and *FCER1A* genes were extremely decreased in practically all IND patients compared to healthy people. The expression of the *CLEC9A* and *CCR5* genes was not detected in almost any individual included in the study.

**Figure 3:**
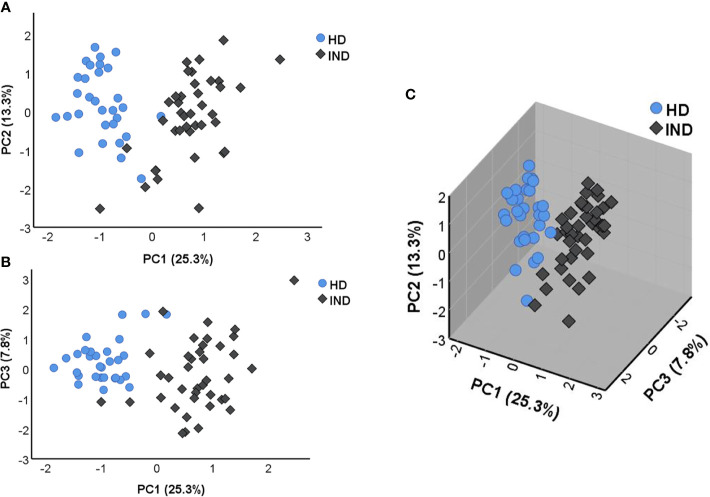
Principal component analysis (PCA) applied on NRQ values of 106 analyzed genes from IND patients (IND, dark rhombus) and healthy donors (HD, blue circles). **(A)** PCA score plot of principal components 1 (PC1) and 2 (PC2) on the x and y axes, respectively. **(B)** PCA score plot of principal components 1 (PC1) and 3 (PC3) on the x and y axes, respectively. **(C)** 3D graphic representing the three principal components 1, 2, and 3 (PC1, PC2, and PC3). The proportion of variance captured for principal components is given as a percentage and indicated on the axis next to the corresponding principal component.

To determine the structure of the data set and examine the variation between the IND and HD subjects, the principal component analysis (PCA) was then performed following the multivariate analysis of the NRQ values. As shown in **Figure 3A**, principal component 1 (PC1) and principal component 2 (PC2) accumulate the largest percentage of the total variance reaching 25.3% and 13.3%, respectively. In addition, principal component 3 (PC3) explains 7.8% of variance, plotted with PC1 in **Figure 3B**. These results together with the 3D graphical representation shown in **Figure 3C** indicate that the level of expression of the genes under study are clearly different between IND and HD subjects which are located on separate groups, mainly based on PC1. The observed differences in PC1 scores between both groups of individuals were confirmed by a two-tailed unpaired t-test which highlighted the existence of a statistically significant different expression profile in IND *versus* HD (*p* < 0.0001). In turn, a two-tail unpaired t-test was applied to the scores obtained for PC2 and PC3, confirming that principal components 2 and 3 did not importantly participate in the differences observed between the two groups of subjects (PC2 *p* = 0.134, PC3 *p* = 0.061).

As shown in **Table 2**, PC1 depends on the expression of 32 genes with the highest factor loadings. Specifically, 23 genes showed a positive correlation with PC1: *FAS*, *IL12RB1*, *BTLA*, *TBX21*, *BCL2*, *CD40LG*, *IL2RG*, *IL12A*, *CD2*, *IL12RB2*, *CD69*, *CASP3*, *IL7*, *STAT1*, *CSF1*, *ICOS*, *CD28*, *CD40*, *TNF*, *IL18R1*, *GATA3*, *IFNG*, and *CD83*), whereas nine genes showed a negative correlation with PC1: *ITGB2*, *CCR1*, *IL18*, *HAVCR2*, *CD86*, *IFNGR1*, *IL17RA*, *IFNGR2*, and *ITGAX.*

**Table 2:** Genes with factor loading of principal component 1 (PC1) higher than 0.6 or lower than -0.6 obtained in the principal component analysis (PCA) including IND and HD subjects.

Gene	Factor Loading for PC1
*FAS*	0.887
*IL12RB1*	0.812
*BTLA*	0.808
*TBX21*	0.808
*BCL2*	0.793
*CD40LG*	0.789
*IL2RG*	0.78
*IL12A*	0.776
*CD2*	0.771
*IL12RB2*	0.755
*CD69*	0.747
*CASP3*	0.74
*IL7*	0.736
*STAT1*	0.734
*CSF1*	0.732
*ICOS*	0.728
*CD28*	0.715
*CD40*	0.705
*TNF*	0.69
*IL18R1*	0.642
*GATA3*	0.619
*IFNG*	0.609
*CD83*	0.608
*ITGB2*	-0.64
*CCR1*	-0.643
*IL18*	-0.691
*HAVCR2*	-0.697
*CD86*	-0.698
*IFNGR1*	-0.736
*IL17RA*	-0.741
*IFNGR2*	-0.759
*ITGAX*	-0.763

**Figure 4:**
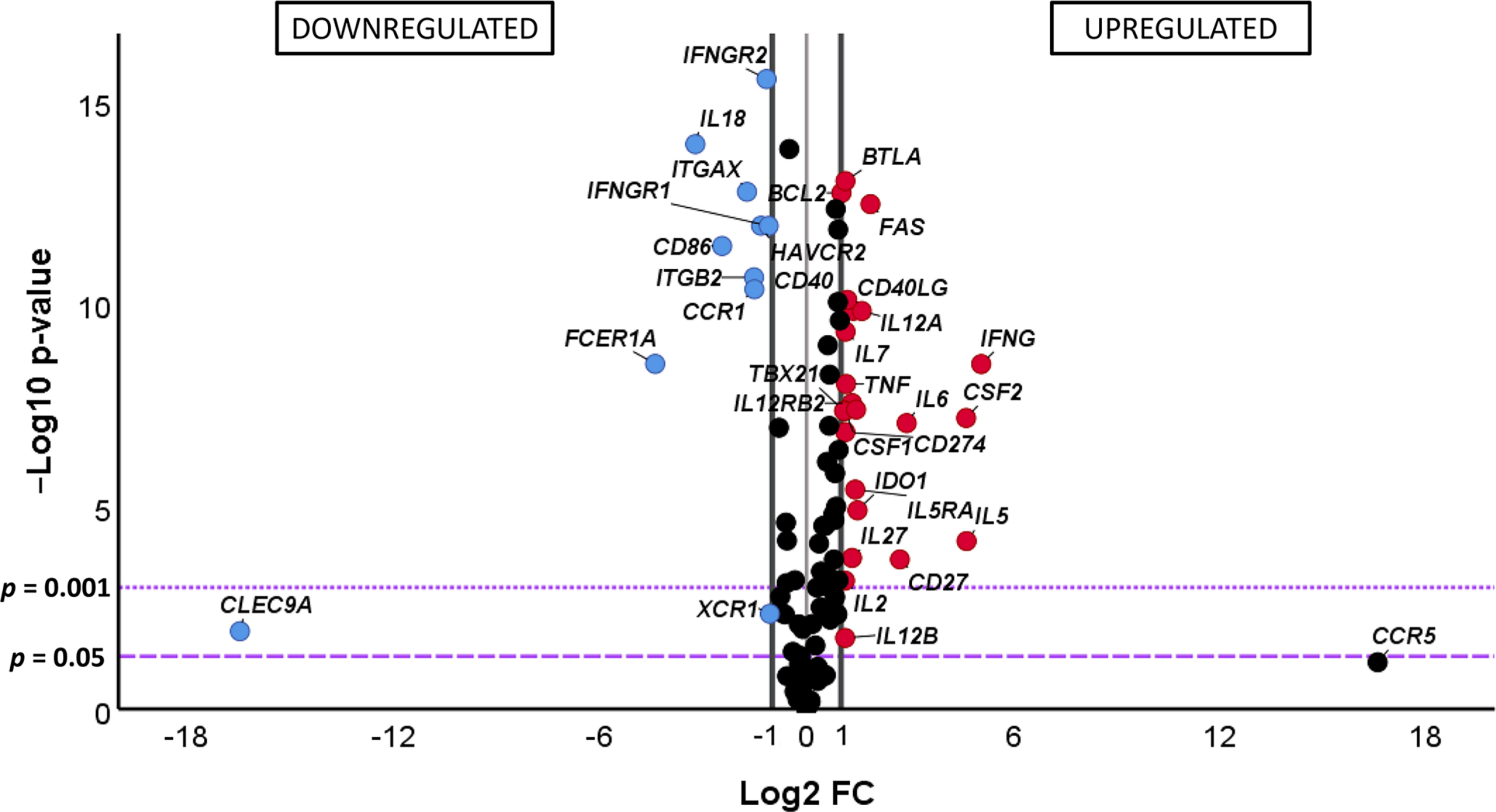
(Comparative) analysis of the differential gene expression level of the 106 analyzed genes in chronic Chagas disease patients with the indeterminate form of the disease (IND) (n = 39) and healthy donors (HD) (n = 30). The x-axis represents log_2_ of the expression fold change between IND and HD (Log_2_ FC), where FC is calculated as the ratio between two groups (IND/HD). The y-axis corresponds to the statistical significance, expressed as the negative logarithm of the *p*-value (-Log_10_ *p*-value). The purple horizontal lines indicate the cutoffs for the statistical significance (corresponding to *p* = 0.05 and *p* = 0.001). The black vertical lines represent the log_2_ FC of −1 and 1 (corresponding to FC of 0.5 and 2, respectively) used as biological thresholds established to identify differentially expressed genes. The negative values correspond to downregulated genes (blue dots) and the positive values to the upregulated genes (red dots) in IND patients compared to HD. Black dots represent non-differentially expressed genes.

In spite of the levels of expression of the genes under study being clearly different between the IND and HD groups of subjects, the no influence of pooling samples of some IND patients was analyzed. For this purpose, PCA analyses were also carried out considering samples from IND patients which came from independent subjects from those which had been mixed as two independent groups. The results, shown in **Supplementary Figure 2A**, revealed that all IND samples maintained the same distribution independently if they came from individual patients (38.5% of the samples) if they had been mixed. The existence of no differences in the gene expression level between the IND patients was also supported by statistical analysis applied to the scores obtained in the two groups for each principal component (**Supplementary Figure 2**). As expected, when these data were compared to those from HD (**Supplementary Figure 2B**), the level of expression of the genes under study showed to be clearly different between IND and HD, as it was previously observed in **Figure 3**. The differential gene expression level in IND *versus* HD of the genes under study was quantified using the fold change of gene expression (FC) as described in *Material and Methods* and FC data and statistical significance represented in a volcano plot. The comparative analysis shown in **Figure 4** indicated that 34 out of 106 genes under study were differentially expressed in IND and HD subjects with statistical significance (-log_10_ *p*-value > 1.3, equivalent to *p*-value < 0.05). Twenty-three of these genes (*BCL2*, *BTLA*, *CD27*, *CD274*, *CD40*, *CD40LG*, *CSF1*, *CSF2*, *FAS*, *IDO1*, *IFNG*, *IL12A*, *IL12B*, *IL12RB2*, *IL2*, *IL27*, *IL5*, *IL5RA*, *IL6*, *IL7*, *STAT1*, *TBX21*, and *TNF*) were significantly upregulated in IND *versus* HD subjects, exhibiting an expression greater than a two-fold change (log_2_ FC > 1, red dots). These differences were statistically significant for all genes, with *p* < 0.001 for 22 out of 23 genes and *p* < 0.05 for the *IL12B* gene. On the contrary, 11 genes (*CCR1*, *CD86*, *CLEC9A*, *FCER1A*, *HAVCR2*, *IFNGR1*, *IFNGR2*, *IL18*, *ITGAX*, *ITGB2*, *XCR1*) showed to be significantly downregulated in IND patients when compared to HD [log_2_ FC < -1, corresponding with less than a half-fold change (blue dots)] with statistical significance in all cases (*p* < 0.05 for *CLEC9A* and *XCR1* genes and *p* < 0.001 for the other nine genes listed in **Figure 4**).

Quantification of the differences observed in the gene expression of the 34 differentially expressed genes in IND *versus* HD was further analyzed considering the log_2_ of fold change values. As shown in the bar plot representation shown in **Supplementary Figure 3**, 34 genes were upregulated at least twice (red bar) or downregulated by half (blue bar) in IND *versus* HD. The *CD27*, *CSF2*, *IFNG*, *IL5*, and *IL6* genes showed the highest differential expression levels as they were overexpressed more than four times (FC > 4) in IND *versus* HD (log_2_ FC values > 2). In addition, the *FAS* and *IL12A* genes showed to be at least three times (FC > 3) upregulated in IND than in HD (log_2_ FC values > 1.5). On the other hand, the expression levels of the *CCR1*, *ITGAX*, and *ITGB2* genes were approximately one-third lower (log_2_ FC values < -1.5, corresponding to FC < 0.35, which means -1/FC = -2.86) in IND compared to HD and the expressions of the *CD86*, *CLEC9A*, *FCER1A*, and *IL18* genes were one-fourth decreased (log_2_ FC < -2, assuming an FC lower than 0.25) in IND patients when compared to the healthy subjects (**Supplementary Figure 3**).

### Analysis of the Differentially Expressed Genes Among Healthy Subjects Coming From Endemic and Parasite-Free Areas of Chagas Disease

**Figure 5:**
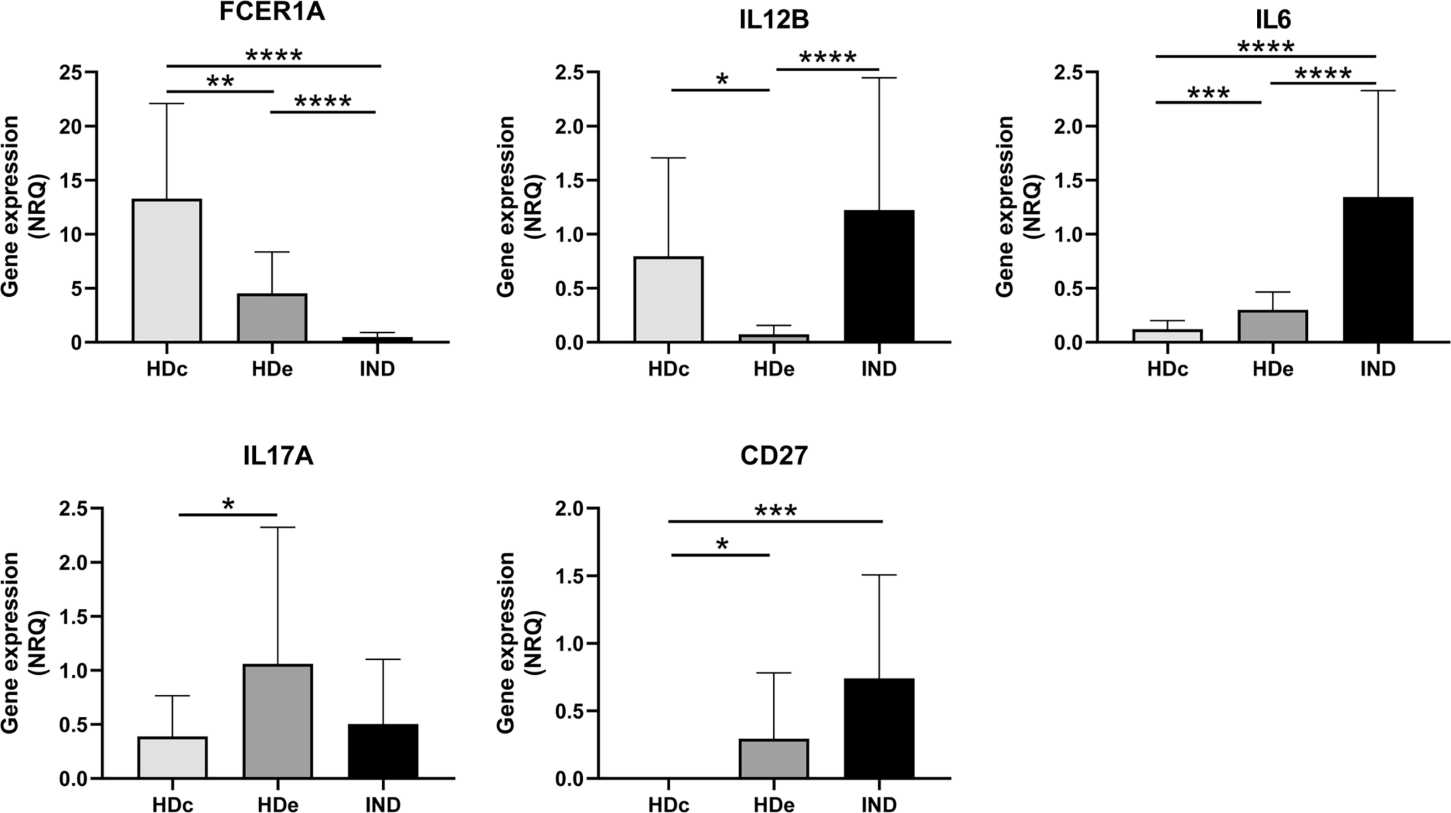
Comparative analysis of the expression levels of *FCER1A*, *IL12B*, *IL6*, *IL17A*, and *CD27* genes measured as mean normalized relative quantities (NRQ) in patients with chronic Chagas disease with indeterminate form of the disease (IND) and healthy donors from endemic (HDe) and non-endemic (HDc) areas of Chagas disease. Statistically significant differences determined by the two-tailed Mann–Whitney test or two-tailed unpaired t-test, as appropriate, are indicated (*p < 0.05, **p < 0.01, ***p < 0.001, and ****p < 0.0001).

Since statistically significant differences in the expression level of five genes (*FCER1A*, *IL12B*, *IL6*, *IL17A*, and *CD27*) were detected between HDc and HDe subjects, we were interested in analyzing in detail how the expression level of these genes in IND patients was and in determining if there was any relationship with that observed in healthy subjects. Thus, comparative analyses of the gene expression levels were carried out for each particular gene among IND, HDc, and HDe subjects. As observed in **Figure 5**, the results showed statistically significant differences in the expression level of these genes among the groups of subjects. Thus, the high expression level of the *FCER1A* gene detected in the HDc subjects (mean NRQ = 13.3) was reduced in the HDe group (NRQ= 4.5) with *p* < 0.01 and particularly diminished in IND patients (NRQ = 0.5) with *p* < 0.0001 when compared to any of the HDc and HDe subjects. The expression of *IL12B* was upregulated in HDc (NRQ = 0.8) *versus* HDe (NRQ = 0.1) with *p* < 0.05 and overexpressed in IND (NRQ = 1.2) with statistical significance when compared to HDe healthy individuals (*p* < 0.0001). Important statistically significant differences (*p* < 0.0001) were also seen in the expression level of the *IL6* gene which was overexpressed in IND patients (NRQ = 1.3) when compared to HDc (NRQ = 0.1) and HDe (NRQ = 0.3). Differences in the expression level of the *IL17A* gene between IND (NRQ = 0.5) and HDc (NRQ = 0.4) or between IND and HDe (NRQ = 1.1) had no statistical significance. Since no expression of the *CD27* gene was detected in HDc, the CD27 level of expression in HDe (NRQ = 0.3) and particularly its overexpression in IND patients (NRQ = 0.7) led to the difference in the level of expression in IND with respect to the HDc that had statistical significance (*p* < 0.001) (**Figure 5**). Altogether, these results suggest that only differences in the gene expression level of the *FCER1A* and *IL6* genes were detected in IND *versus* both HDc and HDe.

### Searching for the Immunological Pathways Implicated in the Establishment of *T. cruzi* Infection in Chagas Disease Patients

To search for immunological routes implicated in the *T. cruzi* chronic infection in Chagas disease patients, the known interactions that take place between the protein-coding genes that showed to be upregulated and downregulated in IND patients when compared to healthy donors were subsequently analyzed. The genes fulfilling both statistical and biological significance thresholds (*p*-value < 0.05 and log_2_ FC > 1 or > -1, respectively) were considered biologically relevant and were used for further biological interpretation. Consequently, a protein–protein interaction (PPI) network was constructed using the STRING platform, requiring the highest confidence in the predicted interactions. The obtained results, plotted in **Supplementary Figure 4**, showed that 34 proteins encoded by the set of differentially expressed genes in IND *versus* HD subjects had a high degree of interaction reaching up to 29 interactions (edges) (from the three expected edges), with a PPI enrichment *p*-value < 1.0e^-16^. Moreover, some of these proteins were grouped according to the most relevant biological pathways in which they are involved based on the pathways belonging to the KEGG and Reactome databases. As summarized in **Table 3**, the results of STRING showed that 6 of these proteins participate in Interleukin-2 family signaling (HSA-451927) with a false discovery rate (FDR) of 5.37e^-09^; 10 proteins in Th1 and Th2 cell differentiation (HSA-04658, FDR 1.3e^-14^); 6 proteins in Interleukin-12 family signaling (HSA-447115, FDR 2.2e^-08^); 14 proteins in Jak-STAT signaling pathway (HSA-04630, FDR 4.77e^-19^); 7 proteins in Th17 cell differentiation (HSA-04659, FDR 3.02e^-09^); and 7 proteins in natural killer cell-mediated cytotoxicity (HSA-04650, FDR 9.75e^-09^).

**Table 3:** Main biological pathways involved in the STRING protein network of differential expressed genes (DEG) between IND and HD subjects.

Database	Pathway	Description	Number of DEG	FDR
Reactome pathways	HSA-451927	Interleukin-2 family signaling	6	5.37e^-09^
KEGG pathways	HSA-04658	Th1 and Th2 cell differentiation	10	1.3e^-14^
Reactome pathways	HSA-447115	Interleukin-12 family signaling	6	2.2e^-08^
KEGG pathways	HSA-04630	Jak-STAT signaling pathway	14	4.77e^-19^
KEGG pathways	HSA-04659	Th17 cell differentiation	7	3.02e^-09^
KEGG pathways	HSA-04650	Natural killer cell-mediated cytotoxicity	7	9.75e^-09^

FDR, false discovery rate.

**Figure 6:**
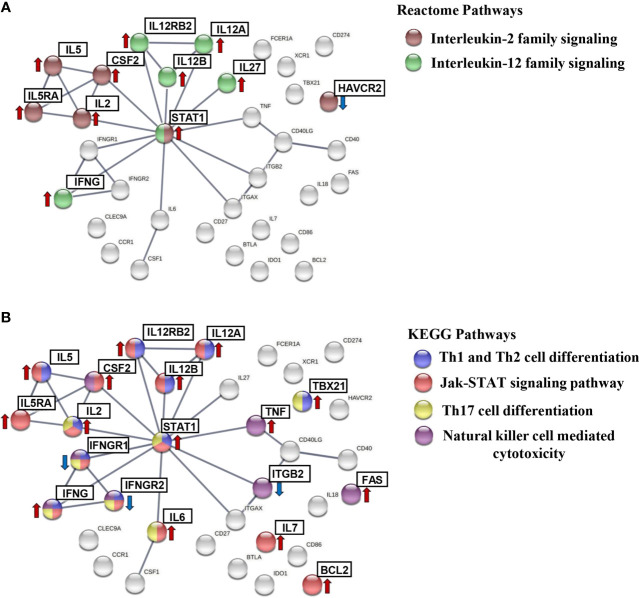
STRING protein–protein interaction (PPI) subnetworks. Representation of the proteins encoded by differentially expressed genes in IND and HD, which are described to be involved in specific pathways. PPI networks were constructed setting the confidence score threshold at the highest level (0.9) and active interaction sources, including data from published experiments, databases, co-occurrence, gene fusion, neighborhood, and co-expression, species limited to “Homo sapiens.” The colored nodes correspond to proteins involved in each biological pathway: **(A)** interleukin-2 family signaling (HSA-451927) (brown) and interleukin-12 family signaling (HSA-447115) (green) from the Reactome Pathways Database and **(B)** Th1 and Th2 cell differentiation (HSA-04658) (blue), Jak-STAT signaling pathway (HSA-04630) (red), Th17 cell differentiation (HSA-04659) (yellow), and natural killer cell-mediated cytotoxicity (HSA-04650) (purple) from the KEGG Pathways Database. The arrows indicate whether the genes encoding these proteins were found to be upregulated (red) or downregulated (blue) in the IND *versus* HD group.

A graphical representation, shown in **Figure 6**, including the aforementioned pathways was obtained in STRING as subnetworks for subsequently highlighting the upregulated and downregulated protein-coding genes in IND patients. As shown in **Figure 6A**, the CSF2, HAVCR2, IL2, IL5, IL5RA, and STAT1 proteins were involved in the IL2 family signaling (**Figure 6A**, brown nodes); IFNG, IL12A, IL12B, IL12RB2, IL27, and STAT1 in the IL12 family signaling pathway (**Figure 6A**, green nodes); IFNG, IFNGR1, IFNGR2, IL12A, IL12B, IL12RB2, IL2, IL5, STAT1, and TBX21 in the Th1 and Th2 cell differentiation route (**Figure 6B**, blue nodes); BCL2, CSF2, IFNG, IFNGR1, IFNGR2, IL12A, IL12B, IL12RB2, IL2, IL5, IL5RA, IL6, IL7, and STAT1 proteins in the Jak-STAT signaling pathway network (**Figure 6B**, red nodes); IFNG, IFNGR1, IFNGR2, IL2, IL6, STAT1, and TBX21 in the Th17 cell differentiation pathway (**Figure 6B**, yellow nodes); and CSF2, FAS, IFNG, IFNGR1, IFNGR2, ITGB2 and TNF proteins included in natural killer cell-mediated cytotoxicity (**Figure 6B**, purple nodes). In all cases, the genes encoding the referred proteins were overexpressed in the IND *versus* HD subjects, with exception of the *ITGB2*, *HAVCR2*, *IFNGR1*, and *IFNGR2* genes which were downregulated.

### Gene Set Enrichment Analysis

**Figure 7:**
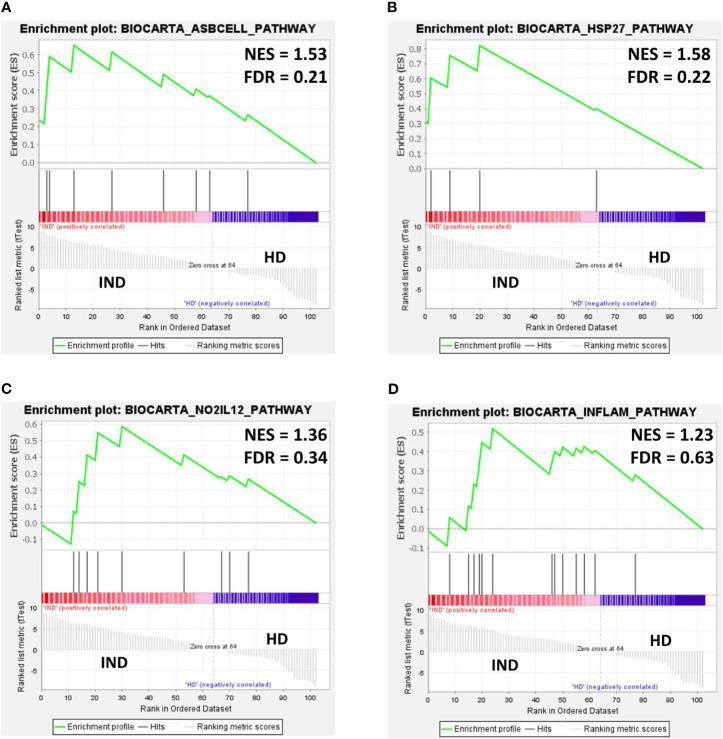
Gene set enrichment analysis (GSEA) plots of representative gene sets from **(A)** antigen-dependent B cell activation pathway (BIOCARTA_ASBCELL_PATHWAY), **(B)** stress induction of HSP regulation (BIOCARTA_HSP27_PATHWAY, **(C)** NO2-dependent IL12 pathway in NK cells (BIOCARTA_NO2IL12_PATHWAY), and **(D)** cytokines and inflammatory response (BIOCARTA_INFLAM_PATHWAY) signature in IND and HD subjects. The green curve denotes the enrichment score (ES) curve. Parameters set for GSEA were as follows: Molecular Signatures Database (MSigDB) BioCarta gene set collection, permutations = 100,000, permutation type: phenotype, enrichment statistic: weighted, metric for ranking genes: t-test, max size: 500, min size: 3. NES, normalized enrichment score; FDR, false discovery rate q-value.

To dissect the immunological pathways associated with the differentially expressed genes in IND *versus* HD, a gene set enrichment analysis (GSEA) was employed using the NRQ values and the Molecular Signatures Database (MSigDB) BioCarta gene set collection (Subramanian et al., 2005; Liberzon et al., 2011). Enrichment plots were obtained from selected pathways to illustrate the positive or negative correlation between the specific gene set upregulated or downregulated in IND and HD for each pathway. As shown in **Figure 7**, GSEA showed a positive correlation in the IND phenotype for several immunological pathways since many genes involved in these routes were overexpressed in IND patients *versus* HD. Thus, the differentially enriched routes in IND showed to be the antigen-dependent B cell activation (BIOCARTA_ASBCELL_PATHWAY, **Figure 7A**), stress induction of HSP regulation (BIOCARTA_HSP27_PATHWAY, **Figure 7B**), NO2-dependent IL12 pathway in NK cells (BIOCARTA_NO2IL12_PATHWAY, **Figure 7C**), and cytokine and inflammatory response (BIOCARTA_INFLAM_PATHWAY, **Figure 7D**) gene sets, with normalized enrichment scores (NES) of 1.53, 1.58, 1.36, and 1.23 plus FDR q-values of 0.21, 0.22, 0.34, and 0.63, respectively.

The number of genes that showed to be upregulated in IND patients was next analyzed, taking into account the proportion of upregulated genes from the total number of genes in the GSEA pathways. To extend the analysis, it was also taken into consideration the identity of the overexpressed genes that showed to have a log_2_ FC higher than 1 and those which participated in principal component 1. As it is observed, **Table 4** integrates the results obtained in GSEA and in PCA and the genes differentially expressed in IND patients *versus* HD for each one of the four BioCarta pathways (with factor loading of PC1 > 0.6 or log_2_ FC > 1). Specifically, 10 out of 15 genes included in the “Antigen dependent B cell activation” BioCarta pathway were analyzed in this study. Five of them (*CD28*, *CD40*, *CD40LG*, *FAS*, and *IL2*) were upregulated in the IND phenotype when compared to HD. From a total of 14 genes that are integrated in the “Stress induction of HSP regulation” BioCarta pathway, we found that four genes (*FAS*, *CASP3*, *BCL2*, and *TNF*) out of five included in the array were overexpressed in IND *versus* HD. Regarding the “Cytokines and inflammatory response” BioCarta pathway, an increased expression of 8 (*IL2*, *TNF*, *CSF1*, *CSF2*, *IFNG*, *IL5*, *IL6*, and *IL7*) out of the 14 genes included in the array from a total 29 genes that are integrated in this route was also detected. In addition, four genes (*IFNG*, *CD2*, *IL12RB1*, and *IL12RB2*) out of nine genes analyzed in the array from the 15 genes included in the “N02-dependent IL-12 pathway in NK cells” pathway were found to be upregulated in IND patients *versus* HD subjects. Of the total of 17 genes that were overexpressed in IND *versus* HD in one or more than one selected pathways, four of them (*CSF2*, *IL2*, *IL5*, and *IL6*) reached a log_2_ of FC > 1 and *p* < 0.05; in the IND *versus* HD subjects, four genes (*CASP3*, *CD2*, *CD28* and *IL12RB1*) had factor loading with PC1 > 0.6 (the principal component responsible for the differences between both groups), and nine genes (*BCL2*, *CD40*, *CD40LG*, *CSF1*, *FAS*, *IFNG*, *IL12RB2*, *IL7*, and *TNF*) fulfilled the criteria for both tests. As expected, the overexpression of these 17 genes in IND *versus* HD was also stated when the same analysis was carried out considering only the samples from IND which came from independent subjects (**Supplementary Table 3**).

**Table 4:** List of genes differentially expressed in IND and HD subjects which have shown to be involved in the BioCarta pathways antigen-dependent B cell activation (BIOCARTA_ASBCELL_PATHWAY), stress induction of HSP regulation (BIOCARTA_HSP27_PATHWAY), cytokines and inflammatory response (BIOCARTA_INFLAM_PATHWAY), and NO2-dependent IL 12 pathway in NK cells (BIOCARTA_NO2IL12_PATHWAY) enriched in IND phenotype according to GSEA analysis.

	Antigen-dependent B cell activation (n = 15)	Stress induction of HSP regulation (n = 14)	Cytokines and inflammatory response (n = 29)	NO2-dependent IL 12 pathway in NK cells (n = 15)		
		
Gene	5 DEG/10 analyzed	4 DEG/5 analyzed	8 DEG/14 analyzed	4 DEG/9 analyzed	Log_2_ FC >1	FL for PC1 > 0.6
***CD28***	**↑**					**×**
***CD40***	**↑**				**×**	**×**
***CD40LG***	**↑**				**×**	**×**
***FAS***	**↑**	**↑**			**×**	**×**
***IL2***	**↑**		**↑**		**×**	
***CASP3***		**↑**				**×**
***BCL2***		**↑**			**×**	**×**
***TNF***		**↑**	**↑**		**×**	**×**
***CSF1***			**↑**		**×**	**×**
***CSF2***			**↑**		**×**	
***IFNG***			**↑**	**↑**	**×**	**×**
***IL5***			**↑**		**×**	
***IL6***			**↑**		**×**	
***IL7***			**↑**		**×**	**×**
***CD2***				**↑**		**×**
***IL12RB1***				**↑**		**×**
***IL12RB2***				**↑**	**×**	**×**

DEG, differentially expressed genes; FC, fold change; FL, factor loading; PC1, principal component 1.

## Discussion

Infection by the *T. cruzi* parasite triggers multiple immune mechanisms in the host to combat the pathogen which can be sustained for decades maintaining the subject in an indeterminate stage of the disease. Along this time, there exists a fragile balance between the replication of the parasite and the host immune response (Dos Santos Virgilio et al., 2014) that, when broken, leads to the progression of the disease. Understanding the molecular mechanisms of pathogenesis and characterizing how the immune system responds to infection result to be essential toward disease control.

In this study, an extensive real-time quantitative PCR (qPCR) analysis has been performed to identify global changes in gene expression profiles of 106 immune system-related genes in IND patients in response to parasite-specific proteins. For comparative analyses, healthy subjects were included in the study taking into consideration the influence of the donor origin, from either endemic or non-endemic areas of Chagas disease. The genes were selected based on their relevance as part of immunological processes which have been described as associated with the control of infection caused by intracellular pathogens. Thus, several genes were selected and analyzed, based on their nature and involvement in biological or immunological functions such as cytokines, chemokines, and their receptors; adhesion molecules; phenotype markers; transcription factors; cytotoxic molecules; inhibitory receptors and their ligands; dendritic cell markers; molecules involved in apoptosis and senescence; costimulatory molecules; and other molecules with immunological involvement.

The first approach was focused on the analysis of the expression profile of the 106 immune-related genes among the healthy donors in response to *Tc*SA stimulation. The expression pattern represented as a heat map together with the principal component analysis failed to differentiate the immune response observed in healthy individuals coming from endemic areas from those of non-endemic regions of Chagas disease. Furthermore, the statistical analyses confirmed that there were no statistically significant differences between the scores of the principal components represented between both groups of subjects (PC1 *p* = 0.65, PC2 *p* = 0.23). On the other hand, differential gene expression analysis revealed that only 4.7% of the genes analyzed (5 out of 106) were differentially expressed between both groups of healthy subjects with statistical significance. These genes were interleukins *IL6*, *IL12B*, and *IL17A* and the phenotype markers *FCER1A* and *CD27*. These findings indicate that there are no differences in the gene expression level of most of the genes under study among healthy donors of different geographical origin. Therefore, all healthy subjects were considered as a single group of subjects in subsequent comparative analyses.

When the gene expression level of 106 immune system-related genes from IND patients was compared to that from healthy subjects, remarkable differences were detected. The NRQ expression values represented in a heat map revealed clear differences in a large group of genes accounting for more than half of the genes under study, with the majority being overexpressed in IND *versus* HD.

The structure of the dataset was determined by PCA analysis, which can be interpreted as a measure of differential gene expression between IND and HD subjects. PCA analysis showed differential gene expression between IND and HD subjects, depending on PC1-correlated genes. The 23 genes positively correlated with PC1 (with factor loading (FL) > 0.6) correspond to cytokines/interleukins and receptors (*CSF1*, *IFNG*, *IL12A*, *IL12RB1*, *IL12RB2*, *IL18R1*, *IL2RG*, *IL7*, and *TNF*), costimulatory molecules (*CD2*, *CD40*, *CD40LG*, *CD69*, *ICOS*), transcription factors (*GATA3*, *STAT1*, *TBX21*), molecules involved in apoptosis (*BCL2*, *CASP3*, *FAS*), one phenotype marker (*CD28*), one inhibitory receptor (*BTLA*), and one dendritic cell marker (*CD83*). Only nine genes showed a negative correlation with PC1 (FL < -0.6) and were cytokines/interleukins and receptors (*IFNGR1*, *IFNGR2*, *IL17RA*, *IL18*), adhesion molecules (*ITGAX*, *ITGB2*), one inhibitory receptor (*HAVCR2*), one dendritic cell marker (*CCR1*), and one costimulatory molecule (*CD86*).

Differential analysis of gene expression revealed that the expression level of a large number of genes varied between infected and non-infected subjects. Specifically, 32% of the genes were expressed more than twice or less than half (log_2_ FC > 1 or < -1) with statistical significance (*p* < 0.05) in IND *versus* HD. For 91% of the genes, very significant differences were detected (*p* < 0.001). The majority of the differentially expressed genes were upregulated in IND (67.6%) particularly cytokines/interleukins and receptors (*CSF1*, *CSF2*, *IFNG*, *IL12A*, *IL12B*, *IL12RB2*, *IL2*, *IL27*, *IL5*, *IL5RA*, *IL6*, *IL7*, and *TNF*), costimulatory molecules (*CD40*, *CD40LG*), transcription factors (*STAT1*, *TBX21*), molecules involved in apoptosis (*BCL2*, *FAS*), inhibitory receptor (*BTLA*, *CD274*), phenotype marker (*CD27*), and enzymes (*IDO1*). The downregulated genes in IND were classified as cytokines/interleukins and receptors (*IFNGR1*, *IFNGR2*, *IL18*), adhesion molecules (*ITGAX*, *ITGB2*), inhibitory receptors (*HAVCR2*), dendritic cell markers (*CCR1*, *CLEC9A*, *XCR1*), phenotype markers (*FCER1A*), and costimulatory molecules (*CD86*).

When the differences observed in gene expression level between IND and HD were quantified, the greatest differences (greater than four times more or less expression in IND *versus* HD) were found for nine genes. Four of these genes showed to be downregulated in IND patients (*CD86*, *CLEC9A*, *FCER1A*, and *IL18*). The expression of the *CLEC9A* gene was not detected in any IND while the *FCER1A*, *IL18*, and *CD86* mRNA levels were 5.5, 22, and 9 times lower in IND than in HD, respectively. The remaining five genes (*CD27*, *CSF2*, *IFNG*, *IL5*, and *IL6*) were upregulated in IND exhibiting the greatest differences (6.5 to 32 times more). It should be noted that the expression of the *IFNG* gene (*IFNγ*) suffered the greatest variation between IND and HD showing an FC value greater than 5, which corresponds to a 32-fold time higher expression in IND patients than in HD. IFNγ is important for orchestrating the development of adaptive immunity, contributing to the differentiation of CD4^+^ Th1 and CD8^+^ T cells required for controlling the parasite proliferation that occurs during acute infection (Cerbán et al., 2020). Thus, the control of *T. cruzi* infection is related to IFN*γ* activation leading to intracellular clearance of parasites (Kulkarni et al., 2015). The expression of the *TNF* gene was also found to be upregulated in IND patients *versus* healthy donors, which is consistent with previous studies that report the detection of high levels of IFNγ and TNF in IND patients (Ferreira et al., 2003; Requena-Méndez et al., 2013), suggesting a relevant role of these molecules in the control of *T. cruzi* infection. In addition to *IFNγ* and *TNF*, the control of *T. cruzi* infection has been associated with the cytokine profile produced by Th1 cells (Petray et al., 1993; Rodrigues et al., 1999; Tarleton et al., 2000; Kumar and Tarleton, 2001; Hoft and Eickhoff, 2005).

Regarding the five genes differentially expressed in subjects coming from endemic and non-endemic areas, three of them (*IL12B*, *IL6*, and *CD27*) were upregulated and one (*FCER1A*) downregulated with statistical significance in IND *versus* HD when they were analyzed as a single group. Although IL17A mRNA was overproduced in healthy donors from endemic areas, the observed differences were not significant when its expression level by IND was compared to that from healthy donors, both as a single group (HD) and as separate groups of individuals (HDc and HDe).

To gain insights into immunological processes to which the differential gene expression profiles observed between IND and HD were associated, a protein–protein interaction (PPI) network was built using the 34 differentially expressed genes using the STRING website. Analysis of the PPI network showed that the differentially expressed genes between IND *versus* HD subjects encode proteins that have a high degree of interaction (PPI enrichment *p*-value < 1.0e^-16^). The most significant biological processes and pathways in which the differentially expressed genes take part were associated with immune response including inflammatory responses, interleukin-2 family signaling, Th1 and Th2 cell differentiation, interleukin-12 family signaling, JAK-STAT signaling pathway, and Th17 cell differentiation and natural killer cell-mediated cytotoxicity pathways. According to STRING analysis, 20 out of 34 differentially expressed genes (59%) were involved in one or more of these six immune system-related pathways with very low false discovery rate (FDR) values (ranging from 4.77e^-19^ to 2.2e^-08^), an indication of the reliability of the predictions.

STAT1 played a central role in five of the highly enriched pathways. The observed *STAT1* upregulation in IND patients supports previous studies that report the activation of STAT1 signaling pathway in host cells after infection with *T. cruzi* leading to a significantly elevated *STAT1* expression (De Avalos et al., 2002). It has been described that *STAT1* plays a major role in the first line of defense against invasion of *T. cruzi* trypomastigotes *(* Stahl et al., 2014 *).* Moreover, it has been reported that STAT1 is a key mediator of IFNγ intracellular signaling and knockout of this protein leads to susceptibility to several intracellular microbes (Kulkarni et al., 2015). The protective effect of *IFN*γ against both the entry of trypomastigotes into host cells and the intracellular multiplication of amastigotes was based on the activation of *STAT1* by tyrosine phosphorylation (Stahl et al., 2014). These results are consistent with the upregulation of both *STAT1* and *IFNG* observed in the present work in IND patients, suggesting the relevant role of these molecules to control *T. cruzi* infection at this stage. Furthermore, upregulation of *IL12A*, *IL12B*, and *IL12RB2* genes has been detected in IND patients, which participate in “Interleukin-12 family signaling,” “Th1 and Th2 cell differentiation,” and “JAK-STAT signaling” according to STRING. IL12 acts on activated T lymphocytes, driving its differentiation to the Th1 subclass. This cytokine is characterized as a potent inducer of *IFNG* production by NK cells and different subsets of T cells (Gately et al., 1994), which is consistent with the differential expression of *IFNG* that we detected in the IND patients. Besides, anti-IL12 antibodies increase susceptibility to infection, highlighting its important role in the control of parasitemia (Aliberti et al., 1996). The results shown here also suggest the possible activation of the “Interleukin-2 family signaling” pathway, detecting an upregulation of this cytokine in IND patients. As it has been reported, *T. cruzi* antigen-specific co-production of IFNγ, IL2, and TNFα by CD8^+^ T cells has been found in a greater proportion in asymptomatic patients, their proportion being decreased according to the progression of the severity of the heart Chagas disease (Lasso et al., 2015; Mateus et al., 2015).

Notably, the differentially expressed genes in IND were also involved in the “Th17 cell differentiation,” as obtained from STRING. Th17 cells correspond to a subset of CD4^+^ T cells known to play a central role in the pathogenesis of many autoimmune diseases, as well as in the defense against some extracellular bacteria and fungi (Ishigame et al., 2009; Lin et al., 2009; Milner et al., 2010; Zielinski et al., 2012). However, their role in intracellular infections has been questioned (Cai et al., 2016). In contrast to this paradigm, the protective role of Th17 cells in the control of parasitemia and survival of *T. cruzi*-infected mice has been reported (Miyazaki et al., 2010; Cai et al., 2016). Moreover, the Th17 profile has been considered a protective factor in preventing myocardial damage in human Chagas disease (Magalhães et al., 2013; Sousa et al., 2017). Our results support this finding and suggest that this pathway may be activated in order to control parasitemia and prevent disease progression in IND patients.

Several gene sets were found to be enriched in IND subjects according to their gene expression levels: “antigen-dependent B cell activation,” “stress induction of HSP regulation,” “NO2-dependent IL12 pathway in NK cells,” and “Cytokines and inflammatory response.” The enrichment was related to the upregulation observed in a number of genes, which were also found either correlated with PC1 (therefore driving the separation of IND and healthy subjects in the PCA analysis) or showing a statistically significant differential gene expression in the volcano plot (FC > 1 and *p* < 0.05). Seventeen genes were included in this classification, of which 52.9% fulfilled both criteria.

Regarding the enriched routes, the activation in these patients of antigen-dependent B cells was a consequence of the upregulation of *CD28*, *CD40*, *CD40LG*, *FAS*, and *IL2* genes. CD40 interaction with CD40L and CD28 interaction with CD80 provide positive costimulatory signals that stimulate B cell activation, proliferation, and differentiation to memory cells (BioCarta—http://www.gsea-msigdb.org/gsea/msigdb/cards/BIOCARTA_ASBCELL_PATHWAY). Inflammation as a protective response to infection is also observed with the enrichment in the “cytokines and inflammatory response” pathway as a result of *IL2*, *TNF*, *CSF1*, *CSF2*, *INFG*, *IL5*, *IL6*, and *IL7* gene upregulation.

The finding that “NO2-dependent IL12 Pathway in NK cells” is enriched in IND patients, who remain asymptomatic and therefore control disease progression, suggests that activation of this pathway may be essential to fight the parasite. A previous study reported that the resistance against *T. cruzi* is based on the release of IL12 by infected macrophages, which induces IFNγ production from T and NK cells (Cerbán et al., 2020). In macrophages, IFNγ functions by activating inducible nitric oxide synthase (iNOS) and NADPH oxidase for the production of nitric oxide (NO), reactive oxygen species (ROS), and reactive nitrogen intermediates (RNI) as peroxynitrite (ONOO-), which are critical for the trypanocidal activity (Gazzinelli et al., 1992; Vespa et al., 1994; Guiñazú et al., 2007).

GSEA analysis also revealed an enrichment in IND patients of the “stress induction of HSP regulation” pathway gene set as a consequence of upregulation of the *FAS*, *BCL2*, *CASP3*, and *TNF* genes included in this route. The activation of these genes, all four involved in apoptosis processes, was expected in Chagas disease patients, given intracellular infection with *T. cruzi*. However, the activation of the “Stress induction of HSP regulation” pathway (BIOCARTA_HSP27_PATHWAY) further suggests that the expression of these genes could be activating heat shock proteins. Heat shock proteins, and particularly Hsp27, have shown to have a strong protective effect on cells, mainly due to its vital function at apoptosis regulation (Garrido et al., 2003; Wang et al., 2014). Interestingly, Hsp27 has shown to have the ability to decrease ROS levels, allowing cells to increase their resistance to oxidative stress (Garrido et al., 1997; Rogalla et al., 1999; Arrigo et al., 2005), so this pathway could also be acting as a mechanism for controlling the presence of ROS in the cell of these patients. This fact seems to result to be essential since it has been shown that when these cytotoxic species are produced in excess or for sustained periods of time or when there is an inadequate antioxidant response, they can accumulate and may contribute to the pathogenesis of Chagas disease (Zacks et al., 2005).

The results shown here indicate that infection with *T. cruzi* induces changes in the expression profile of several genes that seem to be implicated in relevant immunological pathways. These protein-coding genes may result to be useful biomarkers of the indeterminate form of Chagas disease and may act as new therapeutic targets in hosts useful in preventing the progression to the chronic symptomatic phase. The results indicate that the innovative strategy employed here can be applied for future gene expression analyses of more genes involved in the identified pathways and in the identification of new pathways. All this will undoubtedly elucidate the immune response produced in Chagas disease patients and the immunological pathways activated in asymptomatic and symptomatic Chagas disease patients.

## Data Availability Statement

The original contributions presented in the study are included in the article/**Supplementary Material**. Further inquiries can be directed to the corresponding authors.

## Ethics Statement

The studies involving human participants were reviewed and approved by the Ethics Committees of the Consejo Superior de Investigaciones Científicas (Spain—Reference: 094/2016) and of the Hospital Virgen de la Arrixaca (Murcia, Spain—Reference: MTR-05/2016). The patients/participants provided their written informed consent to participate in this study.

## Author Contributions

Conceptualization: ML, EC, and MT. Formal analysis: IG, GP, AE, EC, ML, and MT. Funding acquisition: BV, EC, MSe, ML, and MT. Methodology: IG, AE, GP, BC, MSi, EC, ML, and MT. Writing—first draft: IG. Writing—review and editing: IG, GP, AE, EC, ML, and MT. All authors contributed to the article and approved the submitted version.

## Funding

This work was supported by grant PID2019-109090RB-I00 from the Programa Estatal I+D+I, Spanish Ministry of Science and Innovation (MICIIN), and the Network of Tropical Diseases Research—RICET (RD16/0027/0005, RD16/0027/0001, and RD16/0027/0016).

## Conflict of Interest

The authors declare that the research was conducted in the absence of any commercial or financial relationships that could be construed as a potential conflict of interest.

## Publisher’s Note

All claims expressed in this article are solely those of the authors and do not necessarily represent those of their affiliated organizations, or those of the publisher, the editors and the reviewers. Any product that may be evaluated in this article, or claim that may be made by its manufacturer, is not guaranteed or endorsed by the publisher.
