# Supplementary material for: Differential Expression of Immune Response Genes in Asymptomatic Chronic Chagas Disease Patients Versus Healthy Subjects
Source: Front Cell Infect Microbiol. 2021 Sep 6;11:722984. doi: 10.3389/fcimb.2021.722984 (PMC8450343; doi:10.3389/fcimb.2021.722984)

Supplementary Figure 1. Heatmap of gene expression level of 106 immune-system related genes in healthy subjects from non-endemic (HDc) and endemic (HDe) areas of Chagas disease. The values of the gene expression level of each gene are represented as colors, ranging from red to green, based on the highest and lowest expression level of a gene, respectively.


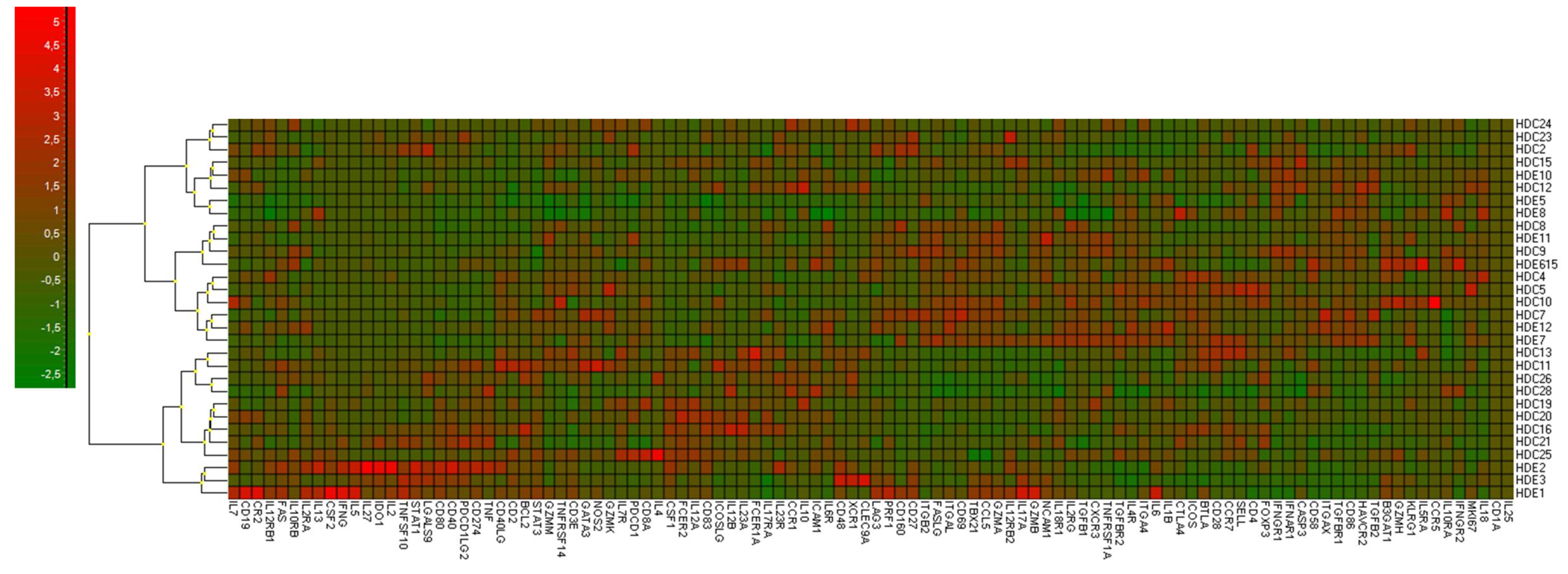

Supplement: Supplementary file 1 [file DataSheet_1.docx]
