# Supplementary material for: Differential Expression of Immune Response Genes in Asymptomatic Chronic Chagas Disease Patients Versus Healthy Subjects
Source: Front Cell Infect Microbiol. 2021 Sep 6;11:722984. doi: 10.3389/fcimb.2021.722984 (PMC8450343; doi:10.3389/fcimb.2021.722984)
Supplement: Supplementary file 2 [file DataSheet_2.docx]

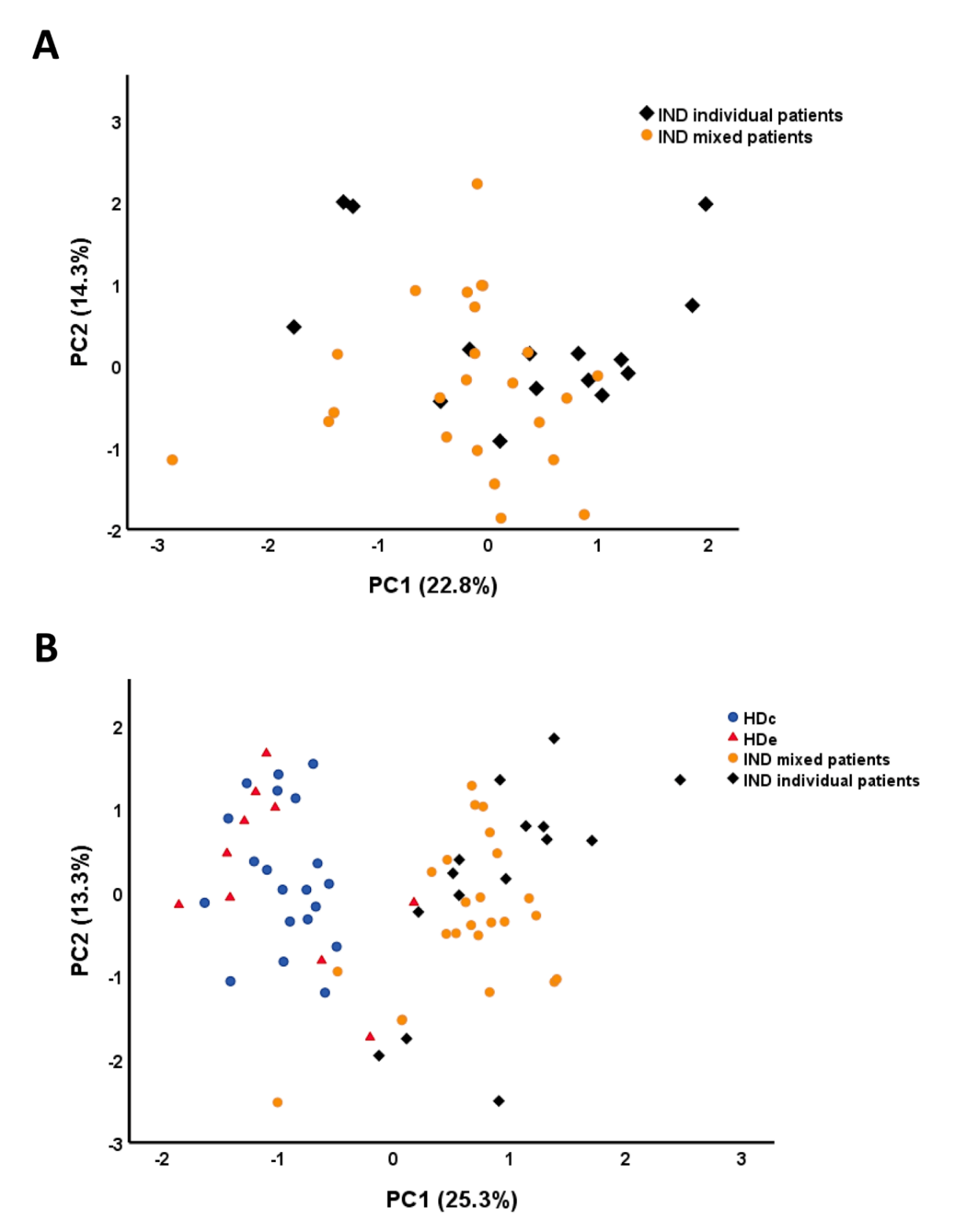


**Supplementary Figure 2.** Principal-component analysis (PCA) of NRQ (Normalized Relative Quantities) values of gene expression of 106 analyzed genes in **(A)** IND mixed patients (orange circles) and IND individual patients (black rhombus) and in **(B)** HDc (blue circles), HDe (red triangles), IND mixed patients (orange circles) and IND individual patients (black rhombus). PC1 and PC2 are plotted on the x and y axes, respectively, and the proportion of variance captured for both components is given as a percentage. These results shown in **(A)** were confirmed by a two-tailed Mann-Whitney test or a two-tailed unpaired t-test, as appropriate, showing that there were no statistically significant differences between the scores obtained in the two groups for each component (PC1 *p* = 0.07, PC2 *p* = 0.09, PC3 *p* = 0.23, PC4 *p* = 0.63).
