# Supplementary material for: Differential Expression of Immune Response Genes in Asymptomatic Chronic Chagas Disease Patients Versus Healthy Subjects
Source: Front Cell Infect Microbiol. 2021 Sep 6;11:722984. doi: 10.3389/fcimb.2021.722984 (PMC8450343; doi:10.3389/fcimb.2021.722984)
Supplement: Supplementary file 3 [file DataSheet_3.docx]

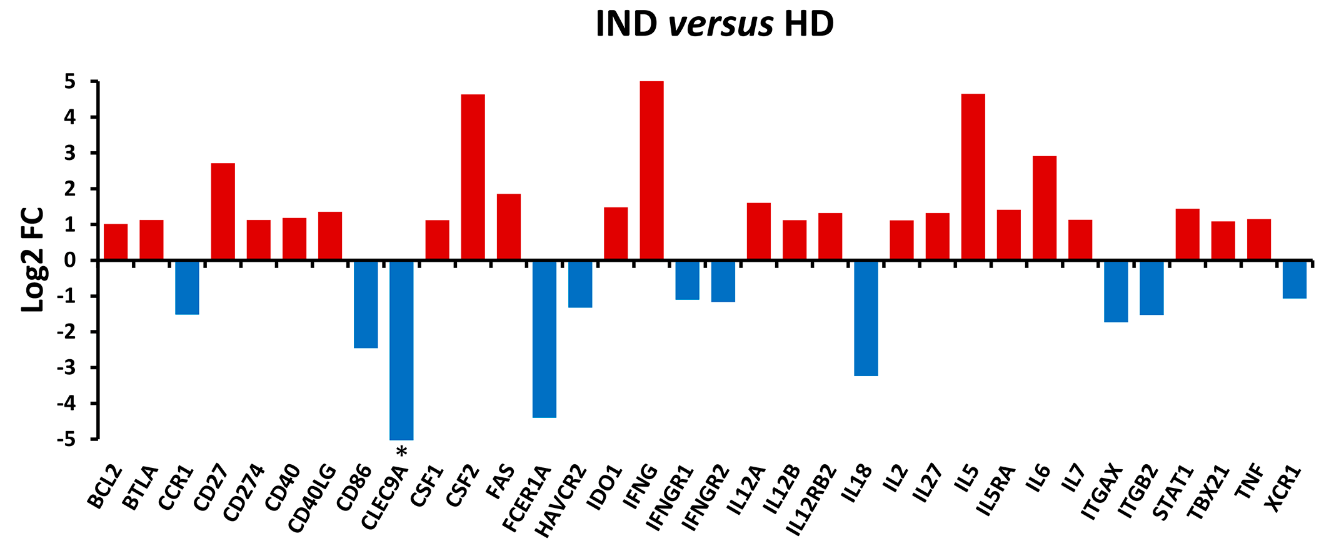


**Supplementary Figure 3**. Analysis of the relative gene expression level of 34 genes differentially expressed with statistical significance in indeterminate patients (IND) *versus* healthy donors (HD). Each bar of the plot corresponds to the gene referred at the bottom of the graph (x-axis). The y-axis represents log_2_ of expression fold change (Log_2_ FC) for each gene. FC is the ratio between the average gene expression of IND and HD groups. Positive values (red bars) indicate upregulated genes and negative values (blue bars) indicate downregulated genes in IND when compared to the HD group. Horizontal gray bars indicate Log_2_ FC = 2 and -2, corresponding with FC of 4 and 0.25, respectively. * Since the *CLEC9A* gene did not show any expression level in the IND group (Log_2_ FC value was -16.47) and to apply the formulas, the value 0 was replaced by 10^-6^.
