# Supplementary material for: Differential Expression of Immune Response Genes in Asymptomatic Chronic Chagas Disease Patients Versus Healthy Subjects
Source: Front Cell Infect Microbiol. 2021 Sep 6;11:722984. doi: 10.3389/fcimb.2021.722984 (PMC8450343; doi:10.3389/fcimb.2021.722984)
Supplement: Supplementary file 4 [file DataSheet_4.docx]

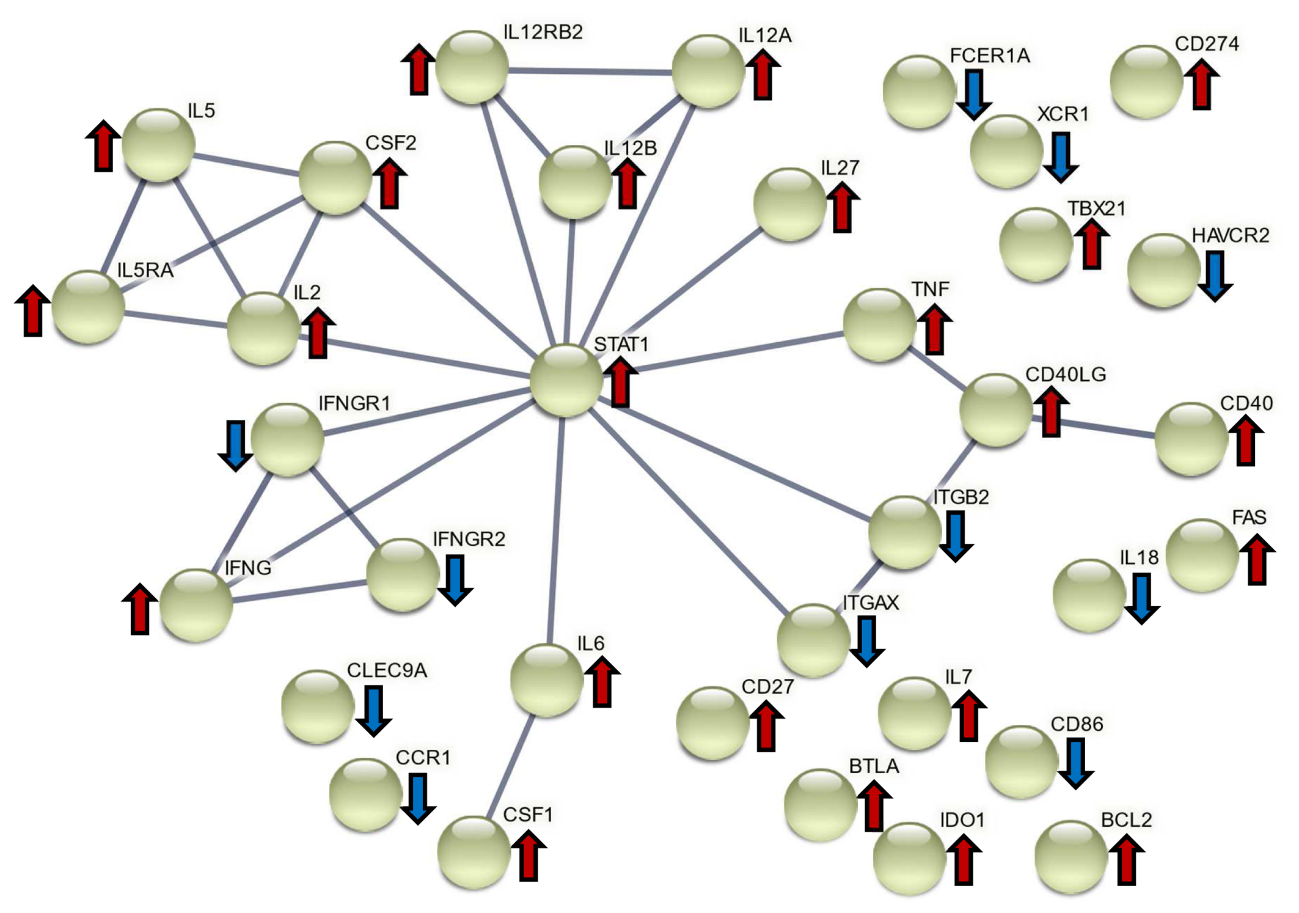


**Supplementary Figure 4**. STRING Protein-protein interaction (PPI) analysis. STRING analysis of the relationship among the 34 differential expressed genes with statistical significance between IND patients and healthy donors (HD). The network nodes represent the proteins encoded by the differentially expressed genes and the linkage of each node represented the interactions (edges) reported for these molecules in different immunological processes; PPI enrichment *p*-value < 1.0e-16. Network edges represent confidence (line thickness indicates the strength of data support). The confidence score threshold was set at Highest (0.9) and active interaction sources, including experiments, databases, co-occurrence, gene fusion, neighborhood and co-expression as well as species limited to “Homo sapiens” were applied to construct PPI networks. The arrows indicate whether the genes encoding these proteins were upregulated (red) or downregulated (blue) in the IND patients *versus* HD.
