# Supplementary material for: Differential Expression of Immune Response Genes in Asymptomatic Chronic Chagas Disease Patients Versus Healthy Subjects
Source: Front Cell Infect Microbiol. 2021 Sep 6;11:722984. doi: 10.3389/fcimb.2021.722984 (PMC8450343; doi:10.3389/fcimb.2021.722984)
Supplement: Supplementary file 5 [file Table_1.docx]

Supplementary Table 1. Main clinical data used for the classification of the IND patients included in the study.

| **Sample ID** | | **ELISA titer** | **IFI titer** | **Cardiac auscultation** | **Thoracic X-ray** | **ECG** | **ECHO** | **Abdominal distension** | **Viscero-megalies** | **Gastro-esophageal transit** | **Opaque enema** |
| --- | --- | --- | --- | --- | --- | --- | --- | --- | --- | --- | --- |
| **1** | | 8.2 | >1/160 | Rhythmic | Normal | SR | Normal | No | No | Normal | Normal |
| **2** | | 6.7 | >1/160 | Rhythmic | Normal | SR | Normal | No | No | Diverticulum | Normal |
| **3** | | 10.4 | >1/160 | Rhythmic | Normal | SR | Normal | No | No | Normal | ND |
| **4** | | 6.9 | >1/160 | Rhythmic | Normal | SR | Normal | No | No | Normal | Normal |
| **5** | | 8.2 | >1/160 | Rhythmic | Normal | SR | ND | No | No | Normal | Normal |
| **6** | | 6.4 | >1/160 | Rhythmic | Normal | SR | Normal | No | No | ND | Normal |
| **7** | | 9.3 | >1/160 | Rhythmic | Normal | SR | ND | No | No | ND | Normal |
| **8** | | 7.2 | >1/160 | Rhythmic | Normal | SR | Normal | No | No | Normal | Normal |
| **9** | | 6.0 | >1/160 | Rhythmic | Normal | SR | Normal | No | No | ND | ND |
| **10** | | 5.5 | >1/160 | Rhythmic | Normal | SR | ND | No | No | Normal | Normal |
| **11** | | 7.2 | >1/160 | Rhythmic | Normal | SR | Normal | No | No | Normal | Normal |
| **12** | | 5.4 | >1/160 | Rhythmic | ND | SR | ND | No | No | ND | ND |
| **13** | | 4.2 | >1/160 | Rhythmic | Normal | SR | Normal | No | No | Normal | Normal |
| **14** | | 4.3 | >1/160 | Rhythmic | Normal | SR | Normal | No | No | Normal | Normal |
| **15** | | 6.6 | >1/160 | Rhythmic | Normal | SR | Normal | No | No | Normal | Normal |
| **16** | **16.1** | 6.9 | >1/160 | Rhythmic | Normal | SR | ND | No | No | ND | ND |
|  | **16.2** | 6.2 | >1/160 | Rhythmic | Normal | SR | Normal | No | No | Normal | Normal |
| **17** | **17.1** | 6.9 | >1/160 | Rhythmic | Normal | ND | Normal | No | No | Normal | Normal |
|  | **17.2** | 6.2 | >1/160 | Rhythmic | Normal | SR | Normal | No | No | Normal | Normal |
| **18** | **18.1** | 8.3 | >1/160 | Rhythmic | Normal | SR | Normal | No | No | Normal | Normal |
|  | **18.2** | 7.8 | >1/160 | Rhythmic | Normal | SR | Normal | No | No | Normal | Normal |
| **19** | **19.1** | 5.6 | >1/160 | Rhythmic | Normal | SR | Normal | No | No | Normal | Normal |
|  | **19.2** | 7.8 | >1/160 | Rhythmic | Normal | ND | Normal | No | No | Normal | ND |
| **20** | **20.1** | 7.7 | >1/160 | Rhythmic | Normal | SR | Normal | No | No | Normal | Normal |
|  | **20.1** | 7.2 | >1/160 | Rhythmic | Normal | SR | Normal | No | No | ND | ND |
| **21** | **21.1** | 7.2 | >1/160 | Rhythmic | ND | SR | Normal | No | No | ND | Normal |
|  | **21.2** | 7.3 | ND | Rhythmic | Normal | SR | ND | No | No | Normal | ND |
| **22** | **22.1** | 1.2 | >1/160 | Rhythmic | Normal | ND | Normal | No | No | Normal | Normal |
|  | **22.2** | 2.4 | >1/160 | Rhythmic | Normal | SR | Normal | No | No | Normal | Normal |
| **23** | **23.1** | 7.7 | >1/160 | Rhythmic | Normal | SR | ND | No | No | Normal | Normal |
|  | **23.2** | 7.7 | >160 | Rhythmic | Normal | SR | Normal | No | No | ND | Normal |
| **24** | **24.1** | 7.3 | ND | Rhythmic | Normal | SR | ND | No | ND | Normal | ND |
|  | **24.2** | 7.5 | >1/160 | Rhythmic | Normal | SR | Normal | Yes | No | ND | ND |
| **25** | **25.1** | 8.6 | >1/160 | Rhythmic | Normal | SR | Normal | No | No | Normal | Normal |
|  | **25.2** | 7.3 | >1/160 | Rhythmic | Normal | SR | ND | No | No | Normal | Normal |

| **Sample ID** | | **ELISA titer** | **IFI titer** | **Cardiac auscultation** | **Thoracic X-ray** | **ECG** | **ECHO** | **Abdominal distension** | **Viscero-megalies** | **Gastro-esophageal transit** | **Opaque enema** |
| --- | --- | --- | --- | --- | --- | --- | --- | --- | --- | --- | --- |
| **26** | **26.1** | 8.6 | >1/160 | Rhythmic | Normal | SR | ND | No | No | Normal | Normal |
|  | **26.2** | 7.6 | >1/160 | Rhythmic | Normal | SR | Normal | No | No | RGE | Normal |
| **27** | **27.1** | 7.2 | >1/160 | Rhythmic | Normal | SR | ND | No | No | ND | Normal |
|  | **27.2** | 6.9 | >1/160 | Rhythmic | Normal | SR | ND | No | No | ND | ND |
| **28** | **28.1** | 8.6 | >1/160 | Rhythmic | Normal | SR | ND | No | No | ND | ND |
|  | **28.2** | 8.2 | >1/160 | Rhythmic | Normal | ND | ND | No | No | Normal | Normal |
| **29** | **29.1** | 8.1 | >1/160 | Rhythmic | Normal | SR | ND | No | No | ND | Normal |
|  | **29.2** | 8.7 | >1/160 | Rhythmic | ND | SR | ND | No | No | ND | ND |
| **30** | **30.1** | 11.1 | >1/160 | Rhythmic | Normal | SR | ND | No | No | ND | ND |
|  | **30.2** | 10.3 | >1/160 | Rhythmic | Normal | SR | ND | No | No | ND | ND |
| **31** | **31.1** | 7.7 | >1/160 | Rhythmic | ND | SR | ND | No | ND | Normal | ND |
|  | **31.2** | 7.9 | >1/160 | Rhythmic | Normal | SR | Normal | No | No | Normal | Normal |
| **32** | **32.1** | 12.3 | >1/160 | Rhythmic | Normal | SR | Normal | No | No | ND | Normal |
|  | **32.2** | 12.9 | >1/160 | Rhythmic | Normal | SR | ND | No | No | Normal | Colon diverticulitis |
|  | **32.3** | 13.2 | 1/180 | Rhythmic | Normal | ND | Normal | ND | No | Normal | ND |
| **33** | **33.1** | 5.7 | >1/160 | Rhythmic | Normal | SR | ND | No | No | Hiatal hernia | Normal |
|  | **33.2** | 5.8 | >1/160 | Rhythmic | Normal | SR | Normal | No | No | ND | ND |
|  | **33.3** | 5.3 | >1/160 | Rhythmic | Normal | SR | Normal | No | No | Normal | Normal |
| **34** | **34.1** | 3.0 | 1/160 | Rhythmic | Normal | ND | Normal | No | No | Normal | Normal |
|  | **34.2** | 3.2 | >1/160 | Rhythmic | Normal | ND | Normal | No | No | ND | ND |
|  | **34.3** | 3.8 | >1/160 | Rhythmic | Normal | SR | ND | No | No | ND | ND |
| **35** | **35.1** | 6.5 | >1/160 | Rhythmic | Normal | SR | Normal | Yes | No | Normal | Normal |
|  | **35.2** | 7.4 | >1/160 | Rhythmic | Normal | SR | ND | No | No | ND | ND |
|  | **35.3** | 6.2 | >1/160 | Rhythmic | Normal | SR | Normal | No | No | ND | Normal |
| **36** | **36.1** | 6.4 | >1/160 | Rhythmic | Normal | SR | Normal | No | No | Normal | Normal |
|  | **36.2** | 6.6 | >1/160 | Rhythmic | Normal | ND | Normal | No | No | Normal | Normal |
|  | **36.3** | 5.7 | >1/160 | Rhythmic | Normal | SR | Normal | Yes | No | Hiatal hernia | Normal |
| **37** | **37.1** | 9.0 | >1/160 | Rhythmic | Normal | SR | Normal | No | No | Hiatal hernia | Normal |
|  | **37.2** | 8.6 | >1/160 | Rhythmic | Normal | SR | ND | No | ND | Normal | ND |
|  | **37.3** | 9.4 | >1/160 | Rhythmic | Normal | SR | Normal | No | No | RGE | Irritable bowel |
| **38** | **38.1** | 4.9 | >1/160 | Rhythmic | Normal | ND | Normal | No | No | ND | ND |
|  | **38.2** | 5.5 | >1/160 | Rhythmic | Normal | SR | ND | ND | No | Normal | ND |
|  | **38.3** | 4.3 | >1/160 | Rhythmic | Normal | SR | ND | No | No | ND | ND |
| **39** | **39.1** | 7.5 | >1/160 | Rhythmic | Normal | SR | ND | No | No | ND | ND |
|  | **39.2** | 6.9 | >1/160 | Rhythmic | Normal | SR | Normal | No | No | Normal | Normal |
|  | **39.3** | 8.3 | >1/160 | Rhythmic | Normal | SR | ND | No | No | Normal | Normal |
|  |  |  |  |  |  |  |  |  |  |  |  |
| **ECG**: Electrocardiogram **ECHO**: Echocardiogram **ND**: not determined **SR**: sinus rhythm **RGE**: gastroesophageal reflux | | | | | | | | | | | |
