# Supplementary material for: Differential Expression of Immune Response Genes in Asymptomatic Chronic Chagas Disease Patients Versus Healthy Subjects
Source: Front Cell Infect Microbiol. 2021 Sep 6;11:722984. doi: 10.3389/fcimb.2021.722984 (PMC8450343; doi:10.3389/fcimb.2021.722984)
Supplement: Supplementary file 7 [file Table_3.docx]

|  | **Log2 FC**  **IND (all samples) *versus* HD** | **Log2 FC**  **IND (samples from individual patients) *versus* HD** |
| --- | --- | --- |
| ***CD28*** | 0.967 | 1.060 |
| ***CD40*** | 1.185 | 1.235 |
| ***CD40LG*** | 1.355 | 1.328 |
| ***FAS*** | 1.856 | 1.898 |
| ***IL2*** | 1.115 | 1.254 |
| ***CASP3*** | 0.846 | 0.930 |
| ***BCL2*** | 1.014 | 1.057 |
| ***TNF*** | 1.148 | 1.227 |
| ***CSF1*** | 1.123 | 1.250 |
| ***CSF2*** | 4.638 | 3.696 |
| ***IFNG*** | 5.076 | 5.510 |
| ***IL5*** | 4.649 | 4.580 |
| ***IL6*** | 2.9099 | 2.041 |
| ***IL7*** | 1.131 | 1.061 |
| ***CD2*** | 0.593 | 0.668 |
| ***IL12RB1*** | 0.660 | 0.725 |
| ***IL12RB2*** | 1.321 | 1.484 |

**Supplementary Table 3.** Comparison of the differential gene expression values obtained for the genes involved in the enriched pathways predicted by GSEA between all IND samples and samples from IND individual patients *versus* healthy donors.
